# Fibrotic pathways and fibroblast-like synoviocyte phenotypes in osteoarthritis

**DOI:** 10.3389/fimmu.2024.1385006

**Published:** 2024-06-04

**Authors:** Alexandra Damerau, Emely Rosenow, Dana Alkhoury, Frank Buttgereit, Timo Gaber

**Affiliations:** ^1^ Department of Rheumatology and Clinical Immunology, Charité – Universitätsmedizin Berlin, corporate member of Freie Universität Berlin and Humboldt Universität zu Berlin, Berlin, Germany; ^2^ German Rheumatism Research Center Berlin, a Leibniz Institute, Glucocorticoids - Bioenergetics - 3R Research Lab, Berlin, Germany

**Keywords:** fibrosis, osteoarthritis, fibroblast to myofibroblast transition, metabolism, mechanical stress, inflammation, senescence, TGF-β

## Abstract

Osteoarthritis (OA) is the most common form of arthritis, characterized by osteophyte formation, cartilage degradation, and structural and cellular alterations of the synovial membrane. Activated fibroblast-like synoviocytes (FLS) of the synovial membrane have been identified as key drivers, secreting humoral mediators that maintain inflammatory processes, proteases that cause cartilage and bone destruction, and factors that drive fibrotic processes. In normal tissue repair, fibrotic processes are terminated after the damage has been repaired. In fibrosis, tissue remodeling and wound healing are exaggerated and prolonged. Various stressors, including aging, joint instability, and inflammation, lead to structural damage of the joint and micro lesions within the synovial tissue. One result is the reduced production of synovial fluid (lubricants), which reduces the lubricity of the cartilage areas, leading to cartilage damage. In the synovial tissue, a wound-healing cascade is initiated by activating macrophages, Th2 cells, and FLS. The latter can be divided into two major populations. The destructive thymocyte differentiation antigen (THY)1^─^ phenotype is restricted to the synovial lining layer. In contrast, the THY1^+^ phenotype of the sublining layer is classified as an invasive one with immune effector function driving synovitis. The exact mechanisms involved in the transition of fibroblasts into a myofibroblast-like phenotype that drives fibrosis remain unclear. The review provides an overview of the phenotypes and spatial distribution of FLS in the synovial membrane of OA, describes the mechanisms of fibroblast into myofibroblast activation, and the metabolic alterations of myofibroblast-like cells.

## 1 Introduction

-Joint inflammation or arthritis is the leading cause of years of disability for millions of people worldwide and is associated with more than 100 types of joint diseases. This heterogeneous group is characterized by the fact that the synovium is the focus of inflammatory activity (1). Herein, fibroblast-like synoviocytes (FLS) have been recognized as key factors in the pathogenesis of the two main types of arthritis: degenerative osteoarthritis (OA) and autoimmune-mediated rheumatoid arthritis (RA) (2). Both diseases have many commonalities. They share pathogenic mechanisms, including leukocyte extravasation, neovascularization, stromal proliferation, and fibrosis-related characteristics. This ultimately leads to micro-lesions within the synovial tissue and structural damage in the joint, including cartilage degradation and bone erosion. These synovial lesions initiate wound healing and tissue repair processes leading to fibrosis features, a pathological condition characterized by an excessive accumulation of extracellular matrix (ECM). The fibrotic state results from the transformation of fibroblasts into myofibroblasts that persist due to insufficient clearance by apoptosis, as opposed to the controlled dissolution observed in normal wound healing. Clinically, patients often suffer from an unrecognized onset of disease, chronicity of disease, repeated flare-ups, joint pain, and joint disability.

However, RA and OA also own distinct pathogenic features. RA demonstrates a higher grade of inflammation driven by autoimmunity and the pannus formation due to hyperplasia of the synovial sublining area. In contrast, OA is considered a degenerative, low-grade inflammatory disease. Herein, OA is a prime example of stress-associated joint disorder due to the constant and intense mechanical strain on the joints (3). The exact mechanisms involved in the transition of fibroblasts into a myofibroblast-like phenotype that drives fibrosis remain unclear.

The aim of this review is, therefore, to (i) briefly summarize current knowledge on the pathogenesis of OA focusing on the (patho-)physiology of the synovial tissue, (ii) provide an overview of possible mechanisms of fibroblast to myofibroblast transformation in OA and (iii) outlines their metabolic alterations that improve our understanding of new potential therapeutic targets. Finally, we present a hypothesis on the development of OA and outline possibilities for future diagnostic and treatment strategies for OA.

## 2 Osteoarthritis: driven by synovitis and fibrosis

Osteoarthritis is a highly prevalent musculoskeletal disorder and the most common form of arthritis. The risk of symptomatic knee and hip OA ─ the two most frequent forms ─ is estimated at 45% and 25%, respectively (4, 5). Due to increasing life expectancy and an active, aging population, OA has become the leading cause of joint pain and age-related disability (3, 6). Among the 369 diseases examined in the Global Burden of Disease Study 2019, OA ranks as the 17^th^ leading cause of disabilities worldwide (6). Consequently, it is predicted that the prevalence of OA will continue to rise, imposing a burden on individuals and healthcare systems. This is evident in the increasing number of joint replacement surgeries (7).

**Figure 1:**
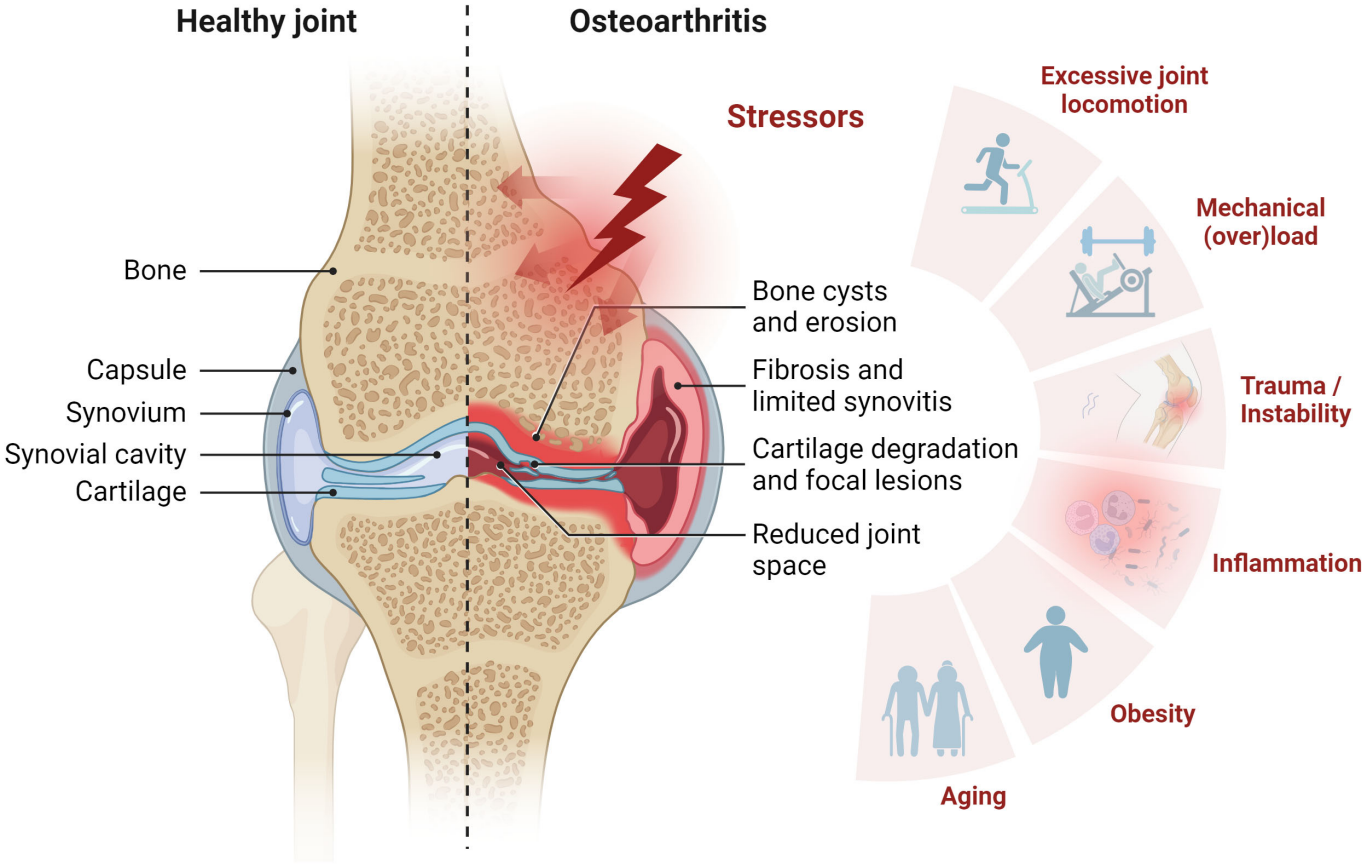
Synovial joint architecture and stressors contributing to osteoarthritis (OA). Schematic representation of healthy joint physiology (left) and OA pathology (right). The joint comprises the femur and the tibia bone, covered with hyaline articular cartilage. To ensure smooth movement, the joint cavity is filled with synovial fluid, which also supplies the avascular cartilage. From the outside, the joint is enclosed by the joint capsule. This consists of an outer fibrous membrane and the inner synovial membrane. OA is characterized by cartilage degradation, bone erosion and bone cysts as well as synovial fibrosis and synovitis. Stress factors contributing to OA include mechanical overload, injury, inflammation, obesity, and aging. Figure was created with BioRender.com.

OA is a chronic, low-grade inflammatory, progressive joint disorder characterized by structural damage to one or more joints (2). Traditionally, OA has been classified as a non-inflammatory joint disease of articular cartilage in elderly individuals (8, 9) due to both the relative lack of neutrophils in the synovial fluid and the absence of a systemic manifestation of inflammation. Nowadays, OA is considered a degenerative whole-joint disease, wherein all components of the synovial joint ─ bone, articular cartilage, synovial membrane, tendon, and ligaments ─ actively contribute to disease progression (**Figure 1**) (10–12). The underlying mechanisms of disease onset and progression are poorly understood, and early diagnosis remains elusive. However, various stressors are known to be associated with or suggested to contribute to OA, including aging, obesity, joint instability, excessive joint locomotion, trauma, and inflammation (**Figure 1**) (13–15). The pathogenesis is characterized by progressive articular cartilage degradation ─ a hallmark of OA ─ partially driven by variable degrees of inflammatory processes, disruption of osteochondral homeostasis, abnormalities of bone contour, osteophytosis, degeneration of knee ligaments, synovitis, hypertrophy of the joint capsule, and fibrosis (3, 10, 16).

The course of OA is highly variable among individuals. OA can affect a single joint or multiple joints, leading to mild or severe joint pain, immobility, and potential loss of joint function, reducing quality of life and considerable socioeconomic burdens (3, 15, 17). Large weight-bearing joints such as hip, hand, knee, and spine are commonly affected (18). Articular cartilage is anatomically capable of responding to the local biomechanical environment, e.g., absorbing and distributing mechanical loads and forming a low-friction system that enables mobility (19). Normally, homeostasis of anabolic and catabolic processes within the joint maintains cartilage integrity by providing lubricants and nutrients, maintaining ECM composition, and removing debris.

In the context of aging, trauma, and OA pathogenesis, mechanical stress and inflammation enhance catabolic processes, driving the breakdown of proteoglycans and collagens. This process, coupled with an imbalanced reactive oxygen species (ROS) production that exceeds antioxidant capacity, culminates in cellular senescence (20–22). Moreover, the composition and organization of the cartilage matrix change while calcification of cartilage ─ a hallmark of OA ─ increases and the synthesis of lubricin declines. The deficiency in cartilage lubrication, which is characterized by the loss of proteoglycans and the breakdown of collagens, is exacerbated by low-grade inflammation of the synovial membrane. This results in an inability of the articular cartilage to properly distribute the mechanical forces within the joints, leading to micro lesion in the synovium (23, 24).

Calcium crystals and breakdown products can trigger an inflammatory cascade in the articular cartilage and the synovial membrane by pattern recognition receptors (PRRs) of the innate response as a part of a sterile tissue injury (23). The PRRs of the toll-like receptor family (TLRs 1-10) are constitutively expressed on cells of the synovial membrane, including macrophages and FLS in OA (25). Notably, FLS can respond to microbial TLR agonists *in vitro* [31, 32]. After TLR engagement, activated FLS produce chemokines (e.g., interleukin (IL)-8)/cytokines (e.g., IL-1, IL-6, and tumor necrosis factor (TNF)-α), thereby recruiting and stimulating additional immune cells (26–28). Infiltration by macrophages is common in both OA and RA and leads to the formation of multinucleated giant cells that enhance phagocytosis (29). Usually, pro‐inflammatory cytokines such as TNF-α, IL-1β, IL-6, IL-8, IL-15, IL-17, IL-21, inflammatory mediators such as prostaglandin E2 (PGE2), nitric oxide (NO), adipokines, matrix metalloproteinases (MMPs) such as MMP-1, MMP-3, MMP-9, MMP-13 as well as aggrecanases, and adhesion molecules such as intercellular adhesion molecule-1 (ICAM-1) and vascular cell adhesion molecule-1 (VCAM-1) are abundant in OA and contribute to its progression (19, 30–32). This disruption of homeostasis results in elevated water content, reduced ECM proteoglycans, a weakening of the collagen network due to decreased synthesis, heightened collagen degradation, increased chondrocyte apoptosis, and is responsible for histological changes in the OA synovium (33).

**Figure 2:**
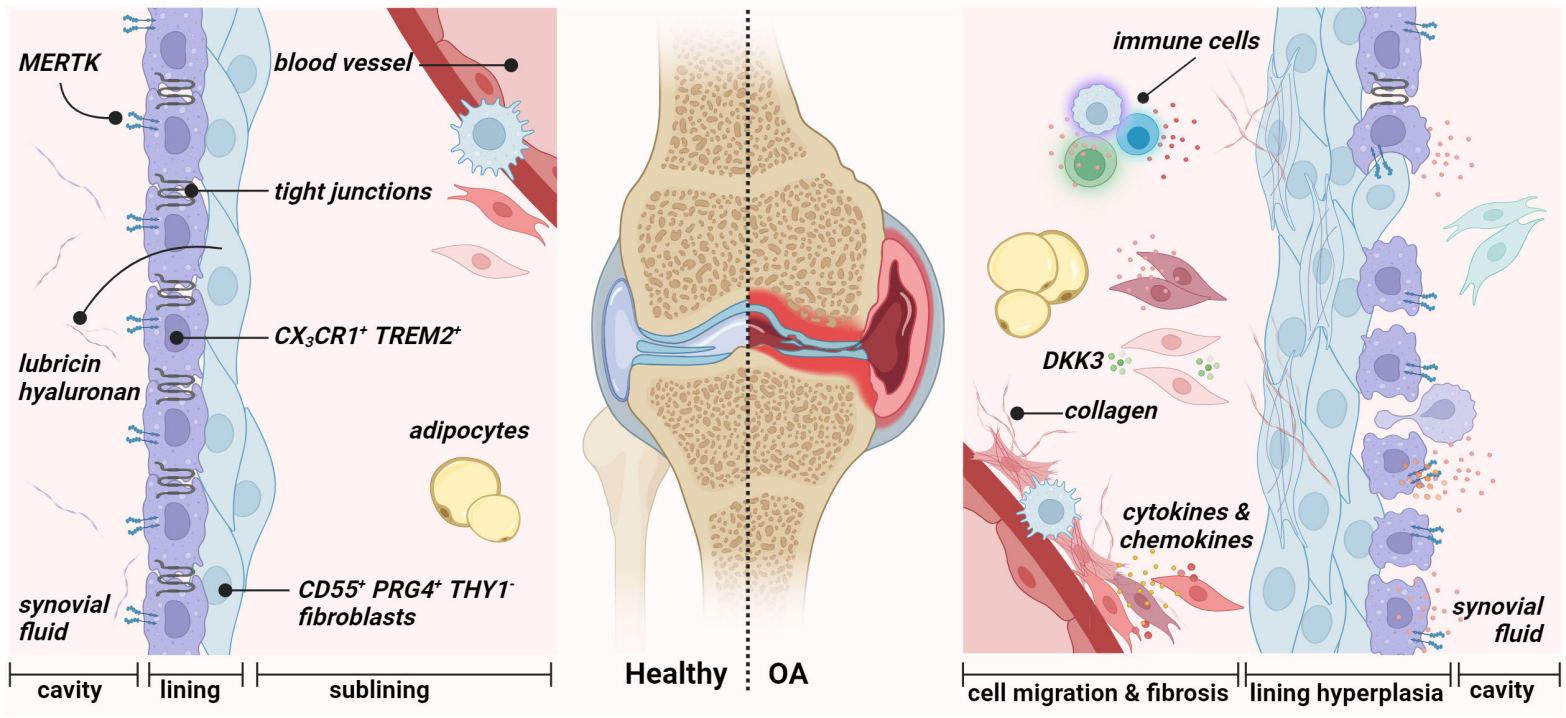
Synovial membrane architecture of the healthy (left) and osteoarthritic joint (right). The healthy synovium comprises a thin lining layer with barrier-forming CX_3_CR1^+^ TREM2^+^ MERTK^+^ resident macrophages (type A synoviocytes) and CD55^+^ PRG4^+^ THY1^−^ fibroblasts (type B fibroblast-like synoviocytes). The lining layer separates the synovial cavity from the tissue. The sublining layer hosts various fibroblast and macrophage populations, adipocytes, and blood vessels. During osteoarthritis, the integrity of the barrier, maintained by tight junctions of resident macrophages, is disrupted in the lining layer. Figure was created with BioRender.com.

The role of synovial fibroblasts in the pathophysiology of OA – crosstalk between synoviocytes and the innate immune system – has been increasingly investigated in the past years. Nevertheless, much is still unknown. The study by Oehler and colleagues in 2002 (34) examined pathologic changes of the synovial membrane in patients with early-stage OA and identified four histological patterns of OA-associated synoviopathy: (i) synovial lining hyperplasia, (ii) sublining and capsular fibrosis, (iii) macromolecular cartilage and bone debris (detritus-rich), and (iv) inflammatory manifestations and stromal vascularization (**Figure 2**) (29, 35–38). Synovitis is known to exist in all stages of OA and is thought to be related to synovial fibrosis in late stages (30, 39). Although the extent of synovitis in OA is, on average, less than in RA (40), significant inflammatory and fibrotic alterations have been found in the synovium of OA patients (41). Previous studies indicate a correlation between the severity and progression of OA with the amount of fibrin deposition and the extent of leukocyte infiltration – with macrophages and T-cells being the predominant immune cells in OA synovium (36). OA is characterized by proliferating FLS, contributing to an imbalance of connective tissue synthesis and catabolism. FLS act as key regulators and promote the production of pro-inflammatory mediators and matrix destructive factors, exacerbating synovial inflammation and initiating a vicious cycle leading to cartilage degradation and joint stiffness ─ characteristic features of OA.

## 3 The physiology of the synovial membrane

Synovium or synovial membrane is the connective tissue that encapsulates the joints without an epithelial or endothelial cell layer. The synovium provides structural support to the joint, lubricates the joint surfaces, especially the cartilage surfaces, and supplies nutrients to the cartilage (42, 43). It lines the inner surface of the joint capsule and the joint cavity and consists of two anatomical and functional layers: the intimal lining (intima) and sublining layer (subintima). In 1962, Barland first reported the successful discovery of two distinct cell types of the lining layer “type A cells” and “type B cells” (44). Since 1996, the term “fibroblast-like synoviocytes” has been used to describe type B cells (45). Finally, in 2011, Smith introduced the terms “type A synoviocytes” and “type B synoviocytes” to describe the two types of synovial cells that occur in relatively equal proportions in the lining layer (16, 43). The synovial lining lacks a basement membrane and tight junctions. It is, therefore, a loose composite of cells embedded in an amorphous matrix composed of collagens such as types I, III, IV, V, and VI (46). In addition, the lining layer is in contact with the intraarticular cavity and responsible for the content of the synovial fluid. Physiologically, it is one to four cell layers thick. The underlying sublining layer, up to 5 mm in thickness, consists of, e.g., fibrous and fatty tissue, blood vessels, and lymphatic vessels and is composed of FLS with fewer macrophage type A cells (**Figure 2**) (43). Type A synoviocytes are non-fixed cells that can actively phagocytose or pinocytose cell debris and waste in the synovial cavity and possess the ability to present antigens. These cells are derived from blood-based mononuclear cells and can be considered resident macrophages (47). Type B synoviocytes are the major stromal cells of the joint synovium that physiologically maintain the structural and dynamic integrity of joints. They can be defined as non-vascular, non-epithelial cells of the synovium that arise during embryogenesis by local division and are replaced by local division (48). Lining layer FLS synthesize matrix components such as hyaluronic acid, collagens, and fibronectin (FN) of the synovial fluid, which performs a lubricating function and allows joint surfaces to slide across each other smoothly (49). Large amounts of hyaluronan are found mainly in the lining layers of normal synovium. These seep into the sublining layer and disappear, indicating diffusion of hyaluronan from the surface toward the clearing lymphatic vasculature.

## 4 The diversity of phenotypes and functions of fibroblast

The joint’s microenvironment is exposed to constant mechanical forces and occasional minor trauma caused by locomotion. In order to maintain homeostasis, this dynamic tissue is constantly being remodeled and repaired by FLS. FLS are not uniform throughout the body. They are specialized in functions depending on their anatomic location (50). Precisely, recent studies using scRNA-seq transcriptomics defined that FLS are a heterogeneous population with several distinct subtypes that exhibit subtype-dependent phenotypic characteristics. Thereby, they differ in their gene expression patterns, epigenetic marks, and functions, which may explain why some joints are more prone to develop certain types of arthritis than others (51). The studies by Stephenson et al. (52) and Mizoguchi et al. (53) have uncovered two major populations of FLS whose localization can be distinguished between lining layer and sublining layer based on the thymocyte differentiation antigen 1 (THY1) expression. These cells from OA joints exhibit a less aggressive cellular behavior than cells from RA joints, meaning proliferation rate, invasive ability, expression, and secretion level of inflammatory cytokines (54).

**Figure 3:**
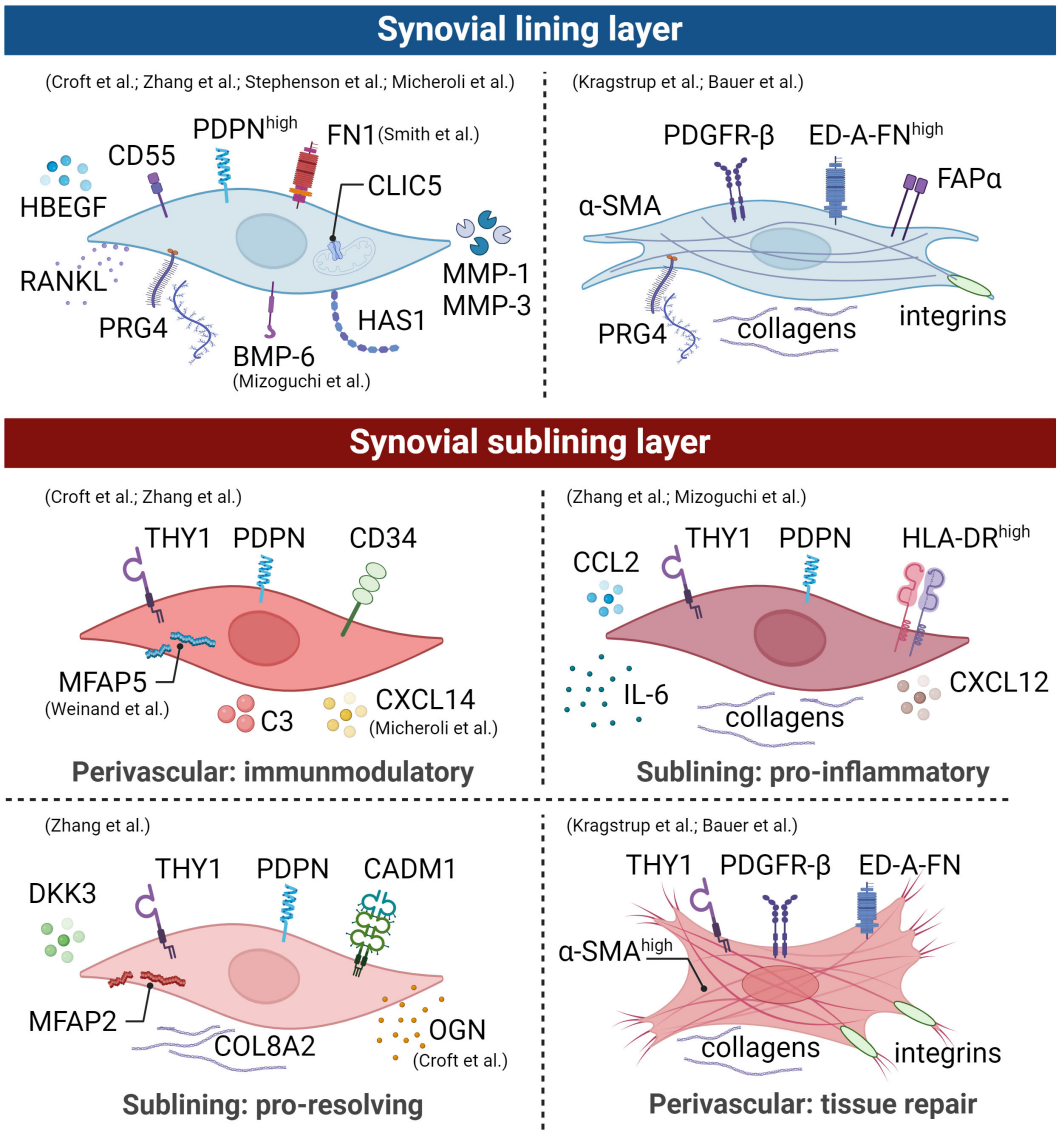
Synovial fibroblast subpopulations are depicted graphically, illustrating surface markers, transcriptional profiles, and functions of distinct subsets within an arthritic joint. The identification of these main subsets involved scRNA-seq studies, mass spectrometry, and histological evaluations. However, it is crucial to emphasize the presence of distinct activation states in human arthritic joints. Therefore, drawing from comprehensive analyses detailed in the text, we propose the existence of an additional lining and sublining layer myofibroblast-like phenotype. Broadly, lining layer FLS lacking thymocyte differentiation antigen 1 (THY1) expression play a pivotal role in lubrication, with this subset being more prominent in osteoarthritis (OA) compared to rheumatoid arthritis (RA). In contrast, sublining layer FLS expressing THY1 expand during inflammation and exhibit different spatial distributions within the joint. This illustration is derived from human data and the figure was created using BioRender.com.

In general, the more aggressive but quiescent podoplanin (PDPN)^+^ CD34^−^ THY1^−^ FLS phenotype is restricted to the synovial lining layer (**Figure 3**). These cells highly express CD55, proteoglycan 4 (PRG4), chloride intracellular channel 5 (CLIC5), FN1, and heparin-binding epidermal growth factor-like growth factor (HBEGF) (51, 55, 56). In this context, CD55 was shown to colocalize with collagen types I and III and with complement C3. It has also been proposed as a protective factor in a mouse model of immune complex-mediated arthritis (57). PRG4 has a known lubricating property, while CLIC5 is localized to the inner mitochondrial membrane and is associated with the modulation of ROS (58). In addition, Stephenson et al. (52) and Mizoguchi et al. (53) reported an increased expression of hyaluronan synthase 1 (*HAS1*) for synovial fluid production and proteases such as MMP-1 and MMP-3, thus contributing to joint homeostasis. Recent research reveals a pronounced higher proportion of CD55^+^ CD34^−^ THY1^−^ FLS in OA compared to RA (53, 55), which might explain the increased bone formation activity in OA since this subpopulation expresses the bone morphogenetic protein (BMP)-6 (53). In addition, this aligns with the fact that OA is characterized by alterations of the synovial lining layer (59). Lining CD34^−^ CD55^+^ THY1^−^ FLS and sublining CD34^–^ THY1^+^ FLS did not differ in their abilities to stimulate osteoclastogenesis via receptor activator of NF-κB ligand (RANKL) and CC motif chemokine ligand (CCL) 9 expression, thereby promoting bone erosion (53).

The THY1^+^ FLS are restricted to the sublining layer and known to act as e.g., invasive, proliferative cells with immune effector function, capable of promoting synovitis. Positive PDPN and THY1 expression characterize three sublining FLS subsets: (1) perivascular CD34^+^ THY1^+^ HLA-DR^low^, (2) pro-inflammatory THY1^+^ CD34^−^ HLA-DR^high^, and (3) THY1^+^ CD34^−^ Dickkopf (DKK)3^+^ HLA-DR^low^ (**Figure 3**) (55, 60). The CD34^+^ THY1^+^ HLA-DR^low^ FLS subset is located within the perivascular region surrounding blood vessels and interacts closely with endothelial cells through NOTCH3 signaling (61). This subset expresses high amounts of complement C3, microfibril-associated protein (MFAP)5, chemokine C-X-C motif ligand (CXCL)14, and genes involved in immune-inflammatory processes and stromal memory (56, 62, 63). Consequently, this FLS subset mediates tissue priming and immunoregulatory function. Zhang et al. performed a subpopulation analysis of synovial fibroblasts isolated from synovial tissue of patients with RA or OA (55). They observed a higher proportion of the second THY1^+^ CD34^−^ HLA-DR^high^ population in RA with increased expression of major histocompatibility complex (MHC) class II, collagens, the interferon (IFN)-γ signaling, IL-6, CCL2, and CXCL12, indicating that these cells are in an active cytokine-producing state (55). The FLS subset THY1^+^ CD34^−^ RANKL^high^ OPG^low^ demonstrates the ability to promote osteoclast differentiation *in vitro* (53, 64). Micheroli et al.’s *in silico* study, integrating an in-house dataset with four published scRNA-seq datasets, affirmed the identification of four distinct fibroblast populations in RA synovial tissue (56). In the sublining layer, CD34^+^ FLS displayed the highest THY1 expression, contrasting with CD34^-^ HLA-DR^high^ FLS, which exhibited the lowest. The third fibroblast subtype, marked by elevated periostin (*POSTN*) expression, demonstrated intermediate THY1 expression (56). Zhang and colleagues showed that this FLS subpopulation expresses high levels of *DKK3*, cell adhesion molecule 1 (*CADM1*), collagens such as *COL8A2*, *MFAP2*, and osteoglycin (*OGN*) (55). By silencing CADM1 in rats with inflammatory bowel disease, Sun and colleagues demonstrated that CADM1 can improve intestinal barrier function (65). Furthermore, Snelling and colleagues investigated the expression of *DKK3* in human OA cartilage, synovial tissue, and synovial fluid (66). They demonstrated that *DKK3* is upregulated in adult human OA cartilage and synovial tissue, decreases during chondrogenesis, and protects against cartilage degradation *in vitro* (66). Overexpression of *DKK3* in FLS upregulated the expression of B-cell lymphoma 2 (BCL2)-associated X apoptosis regulator (*BAX*) promoting apoptosis, suppressed cell proliferation, and reduced collagen synthesis through transforming growth factor (TGF)-β1/Smad2/3 signaling (67). THY1^+^ DKK3^+^ FLS might play a role in immune regulation and restoration of joint homeostasis.

In inflammatory diseases such as acute and chronic arthritis, pathogenic subsets of fibroblasts express the fibroblast activation protein alpha (FAPα). The total number of cells expressing FAPα^+^ THY1^+^ correlates positively with the severity of joint inflammation (51). However, knocking out FAPα expressing fibroblasts in mice did not suppress inflammation but reduced the extent of tissue damage, confirming the pathogenic role of FAPα in arthritis.

Furthermore, the overproduction of ECM proteins and excessive fibrosis can be caused by the activation of a specific subset of fibroblasts called myofibroblasts that can contract wound edges by the contractile protein alpha-smooth muscle actin (α-SMA; encoded by *ACTA2*).

In addition, a subpopulation of fibroblasts known as myofibroblasts produces the contractile protein alpha-smooth muscle actin (α-SMA; encoded by *ACTA2*) that can contract wound edges and contributes to the production of ECM protein (68). Under physiological conditions, these myofibroblasts are eliminated by apoptosis when repair scars form. In certain pathologic scenarios, ACTA2^+^ myofibroblasts persist, leading to excessive fibrosis, a known contributor to the progression of OA (**Figure 3**). Membrane-bound proteins such as platelet-derived growth factor receptor (PDGFR)-β, α11β1 integrin, α5β1 integrin, and αv integrins are frequently found on activated myofibroblasts (69–71). It is important to note that although no specific studies have been performed on synovial myofibroblasts in OA, integrins containing the αv-, α5-, and β1-subunit are increased in the synovial lining layer in OA (72). The αv-subunit forms heterodimers with the β1, β3, β5, β6 or β8 subunits (70). Myofibroblast αv integrins are fundamental elements of a central pathway commonly observed in various solid organs affected by pathological fibrosis.

**Figure 4:**
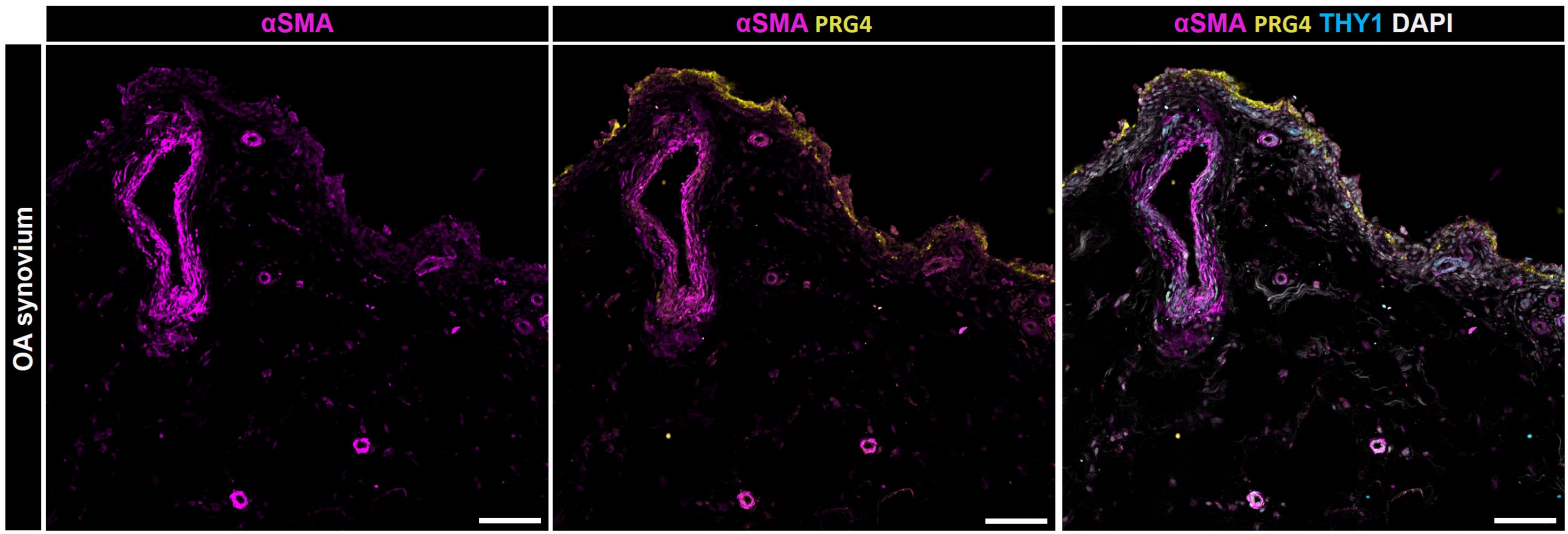
Localization of α-SMA positive cells within the synovial membrane of patients with osteoarthritis. Representative image for PRG4 (yellow), α-SMA (magenta), THY1 (cyan), and DAPI (gray). Scale bars indicate 100 µm (78).

Cell positive for α-SMA^+^ were observed in the synovial membrane in both mice with post-traumatic OA (PTOA) (73) and degenerated anterior cruciate ligaments of patients with OA (74). Hasegawa et al. (74) and Kasperkovitz et al. (75) histologically examined arthritic knee joints and found α-SMA^+^ cells in dense collagenous tissue, in the perivascular area, and the synovial lining layer. The present findings align with those of Bauer and colleagues, who histologically identified a subpopulation characterized by α-SMA^+^ FAPα^+^ FLS within the lining layer and α-SMA^+^ FLS surrounding blood vessels in the sublining layer of both OA and RA synovial tissues (76). Kragstrup and colleagues showed that α-SMA^+^ myofibroblasts isolated from OA synovial tissue also express extra domain A containing fibronectin (ED-A-FN) capable of stimulating macrophage TNF-α production (77). Regarding the localization of α-SMA^+^ cells within the synovium, they further observed a co-localization of ED-A-FN and α-SMA in the OA synovium. While ED-A-FN staining was most intense in lining layer FLS, α-SMA staining was most intense in FLS around the blood vessels. However, most ED-A-FN^+^ FLS in the OA synovial membrane were also α-SMA^+^, demonstrating the presence of myofibroblasts in both the lining and the sublining layer (77). The detection of α-SMA^+^ cells within both the lining and sublining layers aligns with our findings (**Figure 4**). Immunofluorescence staining of OA synovium revealed α-SMA^+^ cells in the lining layer, concurrently expressing PRG4. Additionally, we observed significant α-SMA expression surrounding blood vessels, primarily associated with THY1^+^ cells. This concurrence underscores the heterogeneity of fibroblastic populations in OA synovium and suggests a potential role in maintaining joint homeostasis, vascular remodeling, and synovial pathology.

The *in silico* study by Micheroli et al. identified the third POSTN^+^ FLS subtype, which is most pronounced in a fibroid pathotype and expresses *COL1A1, COL3A1, OGN, TGFB1*, and serpin family E member 1 (*SERPINE1*) (56). Mizoguchi et al. found high expression of *POSTN* and *PDGFRB* in the CD34^−^ THY^+^ subset (53). A spatial atlas illustrating the diversity of synovial fibroblasts in RA was established by Smith et al. (79). Their comprehensive analysis of gene expression in FLS and non-synovial fibroblasts in different tissues and diseases revealed an activated FLS subset (FLS-11) that exhibited transcriptional similarities to ACTA2^+^ myofibroblasts and Wnt family member 5B (WNT5B)^+^ fibroblasts in the colon and two distinct fibroblast populations in scleroderma of the skin. In particular, high expression levels of *POSTN*, *COL3A1*, *NOTCH3*, secreted protein acidic and rich in cysteine (*SPARC*), *THY1*, asporin (*ASPN*), decorin (*DCN*), and *DKK3* were detected in the FLS-11 subset (79). For instance, *COL3A1* was detectable in inflammatory fibroblasts in the colon, myofibroblasts in the lung, and DKK3^+^ synovial sublining FLS, suggesting a potential shared modulatory function of the ECM (80).

In particular, the DKK3^+^ FLS subpopulation is elevated in OA (55). This is consistent with recent studies showing that POSTN is associated with the prevalence, risk of development, and progression of knee OA (81, 82). In addition, the study conducted by Chen et al. revealed a distinctive trajectory in DKK3^+^ FLS within the sublining layer, transitioning from fibroblasts to myofibroblasts (83). This finding implies a possible link between the activation of the DKK3 subpopulation and the differentiation process leading to myofibroblasts in OA (83). However, further studies are needed to investigate the role of fibroblast subsets in synovial tissue fibrosis and to identify their characteristic phenotype. Although the technical innovations of recent years have made tremendous progress, there are still many uncertainties in OA research regarding the spatial arrangement of the various fibroblast subpopulations and the expression of biomarkers that enable early diagnosis of the disease.

In conclusion, examination of the distribution and characteristics of FLS in the synovial sublining and lining layer revealed significant differences between OA and RA. The CD55^+^ THY1^−^ PDPN^+^ CD34^−^ FLS phenotype, which is restricted to the lining layer, is predominant in OA. This subpopulation in OA, which expresses BMP6, may be responsible for the increased bone formation activity observed in this disease. The THY1^+^ FLS are located in the sublining layer and have distinct functional properties, including perivascular, pro-inflammatory and DKK3 expression, each of which is associated with different aspects of synovial pathology. Notably, the DKK3^+^ FLS subpopulation is highly elevated in OA. In addition, α-SMA^+^ myofibroblasts, which are important for tissue repair and fibrosis, have been identified around blood vessels and in the synovial lining layer. This nuanced understanding of FLS phenotypes improves our knowledge on their role in the pathophysiology of OA and may serve as a basis for targeted therapeutic strategies.

## 5 Metabolic pathways in fibroblasts and myofibroblasts

**Figure 5:**
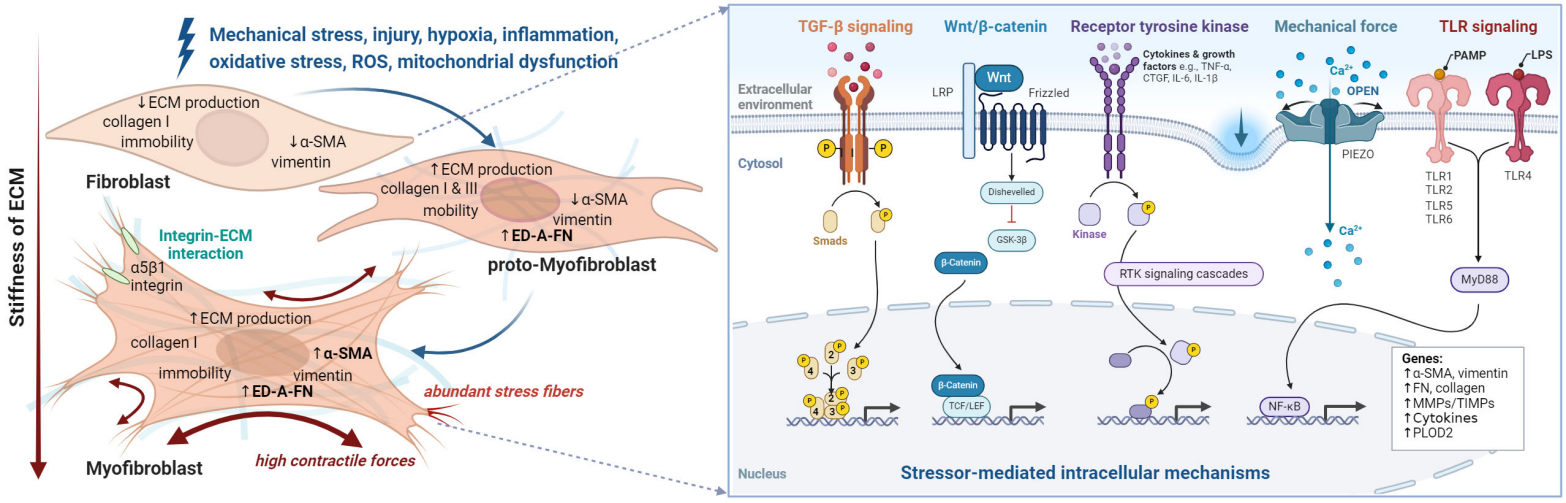
The fibroblast to myofibroblast transition. Schematic overview of stressors and corresponding phenotype alterations (left), and downstream intracellular processes (right) that contribute to myofibroblast differentiation and ultimately lead to synovial fibrosis. Fibroblasts are activated by various types of stimuli such as mechanical and oxidative stress, which initiate different intracellular processes. Initially the proto-myofibroblast develops followed by the myofibroblast. However, activation can sometimes be reversed or lead to apoptosis of the myofibroblasts. At the cellular level, the transition of fibroblast to myofibroblast leads to a pronounced increase in intracellular stress fibers containing alpha-smooth muscle actin (α-SMA) and the expression of collagen 1, extra domain A fibronectin (ED-A-FN), and extracellular matrix remodeling enzymes. Moreover, they produce cytokines such as transforming growth factor (TGF)-β, vascular endothelial growth factor (VEGF), connective tissue growth factor (CTGF), interleukin (IL)-1, IL-6, and IL-8 and are in close contact with their environment via integrins. Figure was created with BioRender.com.

Fibrosis results from a dysregulated wound-healing process characterized by transitioning from mesenchymal cells, such as fibroblasts, into myofibroblasts and excessive deposition of ECM components (77, 84, 85). Fibrosis leads to tissue scarring that can impair organ function in many chronic diseases, including pulmonary fibrosis, liver cirrhosis, and systemic sclerosis, but also in musculoskeletal diseases, such as RA and, more importantly, OA (86, 87). At the cellular level, fibroblasts play a central role in maintaining tissue homeostasis and responding to stressors. Under prolonged injury, inflammation, or mechanical stress, fibroblasts can phenotypically transform into myofibroblasts characterized by α-SMA expression, synthesis of collagen-rich ECM, and fibroblast proliferation (88). Not all activated fibroblasts necessarily transform into myofibroblasts (89). The transition from fibroblasts to myofibroblasts is a dynamic and reversible process driven by multiple signals. Among the most important are (i) the TGF-β signaling pathway that triggers downstream events such as the expression of connective tissue growth factor (CTGF), (ii) pathological mechanical forces (mechanotransduction) that occur through overload, repetitive stress or injury, (iii) pro-inflammatory cytokines and innate immunity in a persistent inflammatory environment, and (iv) excessive oxidative stress leading to cellular aging and senescence (**Figure 5**) (90). In the context of OA, joints’ constant and intense mechanical stress, high TGF-β levels, and low-grade inflammation create an environment that promotes fibroblast activation and transition into myofibroblasts, as explained below.

### 5.1 Crosstalk among TGF-β/Smad-signaling pathway, integrins, and ECM

The pleiotropic cytokine TGF-β is a multifaceted regulator that affects fundamental biological processes such as cell division, proliferation, apoptosis, and tissue homeostasis. Three isoforms of TGF-β are known so far. TGF-β is stable as a non-covalently bound complex with the latency-associated peptide (LAP). This complex is associated with latent TGF-β binding protein (LTBP) via disulfide bonds and is stored within the ECM post-secretion (91, 92). Various factors and conditions, including bioactivation through integrins, mechanical forces, and acidic pH, can trigger the activation of TGF-β *in vitro* (92–95).

Integrins, transmembrane proteins consisting of an α-subunit and a β-subunit, mediate communication between the ECM and fibroblasts. They play a crucial role in the dynamics of tissue fibrosis (70, 72). By connecting the inner cytoskeleton to the ECM, integrins contribute to important catabolic reactions for ECM degradation (70). Despite limited data on the interaction between integrins and OA FLS, differences in integrin expression were observed between the lining and sublining layers of the synovium. In particular, increased expression of integrins such as α5, αv and β1 were found in the lining layer compared to the sublining layer (96). Furthermore, there is convincing evidence that lining layer FLS in OA consistently show strong expression of the integrin subunit αv, while being more diverse and heterogeneous among synovial lining FLS in RA (97).

Upon binding to αv integrins on adjacent cells, the latent complex releases the captive TGF-β through mechanisms specific to the types of αv integrins on the given cell. Integrins αvβ6 and αvβ8 on fibroblasts activate TGF-β1 by binding the RGD-motif on LAP and inducing a conformational change (98, 99). By blocking αv integrins with the small molecule CWHM 12, it is possible to inhibit TGF-β1 signaling and achieve the desired anti-fibrotic effect (69). In OA FLS, integrin αvβ6 plays a critical role due to its upregulated expression upon TGF-β1 stimulation and in mediating the activation of TGF-β. Furthermore, the vitronectin fragment VTN_(381-397 a.a.)_, highly abundant in the serum of OA patients, competes with TGF-β1 for integrin binding and can attenuate TGF-β1 activation (100). Fibronectin can bind to integrins such as α5β1, ανβ3, ανβ5 (101) and ED-A-FN is associated with myofibroblasts and OA (77). Injection of fibronectin fragments into rabbit joints is now an established animal model of OA, characterized by cartilage degradation and osteophyte formation (77, 102). Kragstrup further showed that ED-A-FN is produced in response to TGF-β and self-induces the production of TNF-α in macrophages (77), suggesting ED-A-FN as an interesting target for therapeutic intervention to reduce pro-inflammatory responses in OA.

**Figure 6:**
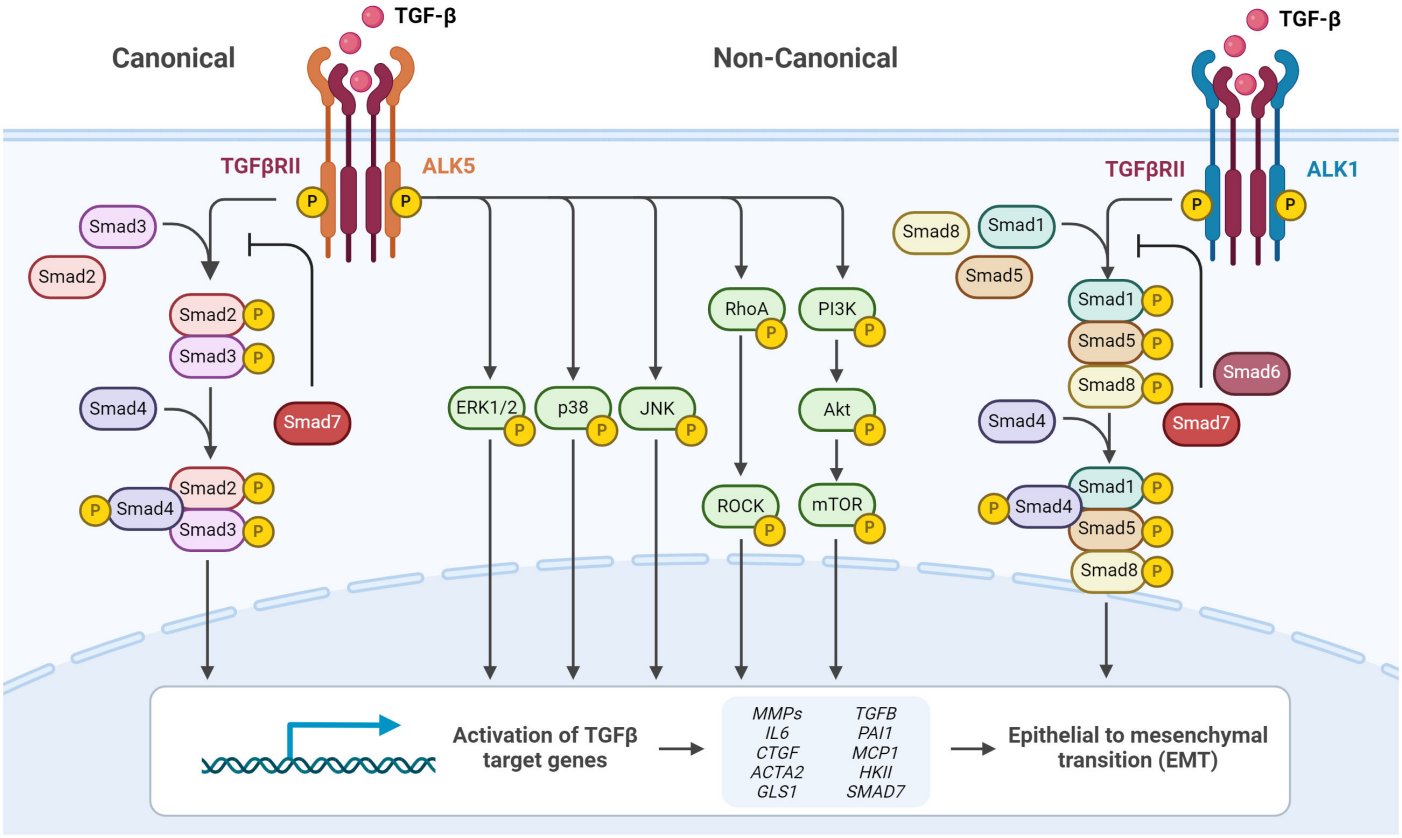
TGF-β signaling via canonical and non-canonical pathways. Cell surface TGF-β type II receptor binds soluble active TGF-β, which causes association and phosphorylation of TGF-β type I receptor. TGF-β type I receptor/ALK5 activation can induce the canonical phosphorylation of Smad2/3 signaling or non-canonical signaling via non-Smad pathways by phosphorylation of, e.g., ERK1/2. Alternatively, TGF-β type I receptor/ALK1 activation can induce the non-canonical phosphorylation of Smad1/5/8. Phosphorylated Smad2/3 and Smad1/5/8 form complexes with Smad4, which translocate into the nucleus and regulate target gene expression. Inhibitory Smad6 and Smad7 can repress canonical or non-canonical signaling via Smad. Figure was created with BioRender.com.

The initiation of the canonical TGF-β signaling pathways involves β-glycan serving as a co-receptor to present TGF-β1 to transmembrane serine/threonine kinase type I and type II receptors (TGFBR1 and TGFBR2, respectively) (103). The receptor complex formation upon TGF-β1 binding by TGFBR2 leads to the phosphorylation of TGFBR1 (ALK5), which in turn phosphorylates Smad2 and Smad3 forming a complex with Smad4 (**Figure 6**). The trimeric complex translocates to the nucleus and regulates different target gene expressions in concert with transcriptional co-activators and co-repressors. One of these target genes is the myofibroblast marker α-SMA (104, 105). Smad signaling is attenuated by inhibitory Smads (I-Smads, Smad6 and Smad7), dephosphorylation and dissociation from the trimeric complex, allowing for either Smad recycling or degradation (106).

Apart from the canonical TGF-β1/Smad2/3-signaling TGF-β can signal via non-canonical pathways. Upon TGF-β stimulation, ALK1 another TGFBR1 forms a complex with ALK5, leading to Smad1/5 activation (107). Furthermore, it has been demonstrated that TGF-β activates a number of other non-canonical (non-Smad) signal pathways in different cell types, such as mitogen-activated protein kinases (extracellular signal-regulated kinase (ERK), c-Jun N-terminal kinase (JNK), and p38), phosphatidylinositol-3-kinase, and GTPases that resemble Rho (**Figure 6**) (108).

RA synovial fluid, rich in TGF-β, has been demonstrated to induce α-SMA expression in adipose-derived mesenchymal stromal cells (MSCs) via Smad2 signaling, indicating TGF-β’s pivotal role in fibrotic phenotype progression (109). Moreover, the deletion of Tgfβr1 prevented TGF-β-mediated differentiation (110) whereas a constitutively active TGFBR1 mutant promoted myofibroblast formation (111) confirming the importance of the canonical signaling in myofibroblast differentiation. Furthermore, inhibition of TGF-β-mediated activation of ALK4/5/7 in osteoarthritic human FLS prevented the induction of the pyridinoline cross-linking enzyme pro-collagen lysine-2-oxoglutarate 5-dioxygenase 2 (*PLOD2*), which suppresses collagen degradation, as well as *CTGF* and *COL1A1*. In contrast, inhibition of ALK1/2/3/6 blocked the induction of *CTGF* and *COL1A1* (112). However, the non-canonical TGF-β-mediated signaling cascades are also implicated in fibrosis. In patients with the fibrotic disease systemic sclerosis, again TGF-β-dependent upregulation of *COL1A1* and *CTGF* is conveyed via ALK1/Smad1/5- and also ERK1/2-signal pathways rather than ALK5/Smad2/3-activation (113). The activation of the aforementioned non-canonical TGF-β-mediated signaling cascades have been implicated in the pathogenesis of fibrosis and fibrotic diseases including renal fibrosis, liver fibrosis, systemic sclerosis and osteoarthritis (107, 108).

In cells from articular cartilage, synovial tissue and fluid from patients with OA, elevated levels of the transcription factor hypoxia inducible factor (HIF)-1α were found, indicating hypoxic conditions, that are associated with progressive joint damage (114, 115). Once hypoxia is established in the tissue microenvironment, cellular HIF-1α is not hydroxylated and therefore forms a heterodimer with HIF-1β initiating a cellular adaptive program by inducing the transcription of genes such as vascular endothelial growth factor A (*VEGFA*), *TGFB*, insulin-like growth factor 2 (*IGF2*), collagen type 2 alpha 1 (*COL2A1*), and aggrecan (*ACAN*). This adaptive transcriptional program promotes angiogenesis, shifts the metabolic program towards glycolysis and maintains ECM homeostasis (116, 117). Hypoxia-mediated TGF-β1 expression led to the release of IL-1β and further induces TGF-β1, suggesting a positive self-enhancing feedback loop between inflammation and fibrosis during myofibroblast activation. This emphasizes the importance of studying synovial fibroblasts in the context of OA with a focus on hypoxia. Boer et al. performed a genome-wide association analysis for OA across 21 cohorts (118). They investigated differential gene expression, methylation or protein abundance in osteophytic cartilage compared to low-grade (intact) cartilage. This study identified genes that are important in TGF-β signaling and function and that fibrosis is a major contributor to the degenerative changes in OA (118). Additional fibrosis-associated genes activated by TGF-β in OA include ECM-encoding genes *COL1A1, COL3A1, COL5A1, FN1*, and key enzymes important for collagen synthesis such *PLOD2*, prolyl 4-hydroxylase subunit alpha 3 *(P4HA3*), and lysyl oxidases (*LOXs*) (91, 106, 119). In FLS from OA patients stimulated with TGF-β, Remst et al. further observed an upregulation of the tissue inhibitor of metalloproteinase-1 (*TIMP1*), indicating inhibition of MMPs (120). Furthermore, exposure of OA FLS to TGF-β has been shown to upregulate PRG4 (121). This molecule also plays a distinct role in myofibroblast differentiation and in the expression of pro-fibrotic genes (122). For *in vitro* studies, various groups have utilized a broad range of TGF-β concentrations over different time intervals, as summarized in **Table 1**. The pathophysiological concentrations of active TGF-β in the synovial fluid of patients with OA and RA were determined to be 4 ng/mL and 10 ng/mL, respectively (129, 130). However, after acidic activation, increased concentrations of up to 12 ng/mL and 37.5 ng/mL were detected in the synovial fluid of OA and RA patients, respectively, indicating the presence of higher concentrations of latent TGF-β (129).

**Table 1:** Summary of TGF-β concentrations used in different studies in the context of synovial fibroblasts and/or osteoarthritis (OA)/rheumatoid arthritis (RA).

TGF-β [ng/mL]	Time interval	Cell type	Ref’s
0.2	24 h, 48 h, 96 h	Human adipose-derived mesenchymal stromal cells	(109)
1	24 h	OA synovial fibroblasts	(121)
5	24 h	Healthy, OA and RA synovial fibroblasts	(123)
10	24 h, 48 h, 72 h	RA synovial fibroblasts	(124)
10	3 weeks	Cell suspension from OA synovium or RA FLS with CD14^+^ monocytes	(125)
10	4 h	RA synovial fibroblasts	(126)
10	6 days	RA synovial fibroblasts	(127)
10	3 or 7 days	OA synovial fibroblasts	(100)
20	30 min or 24 h	RA synovial fibroblasts	(128)
1, 3, 10, 30	24 h	OA synovial fibroblasts	(37)

### 5.2 Role of the CTGF signaling pathway in OA synovial fibrosis

The TGF-β target CTGF, also known as CCN2, plays a crucial role in physiological and pathological processes such as inflammation, wound healing, tumorigenesis, and fibrosis (131). Several factors such as TGF-β (120), macrophage-colony stimulating factor (M-CSF) (132), VEGF (133), PGE2 (134), and mechanical stressors (135, 136) induce high expression of CTGF. However, CTGF can function independently and promote a positive feedback loop itself by increasing e.g., TGF-β, VEGF, and integrins (137). Along with TGF-β, CTGF is abundantly expressed in the synovial fluid of OA (138, 138) and RA patients (139) positively correlating with disease severity. The transfection of the synovial lining layer of mice with an adenovirus expressing human CTGF exacerbated synovial fibrosis, as evidenced by increased pro-collagen type 1 levels, accumulation of ECM, and depletion of proteoglycans in articular cartilage (140). Cartilage degradation may be triggered by CTGF expression or CTGF-mediated cytokine secretion from fibrotic synovial tissue. Davidson et al. further demonstrated that CTGF-induced fibrosis is transient (140), in contrast to the relatively persistent fibrosis induced by TGF-β (39). Liu and colleagues showed that stimulation of OA FLS with CTGF induced concentration-dependent production of the pro-inflammatory cytokine IL-6 via the CTGF-αvβ5-JNK pathway (141). They further revealed that CTGF can trigger the migration of monocytes in OA by enhancing monocyte chemoattractant protein-1 (MCP-1) expression through interaction with integrin avβ5 (142). The study by Yang et al. reported that CTGF increased the activity of focal adhesion kinase (FAK), mitogen-activated protein kinase kinase (MEK), and ERK proteins (143), which is consistent with the findings of Tan and colleagues that CTGF promotes chondrosarcoma cell migration by increasing the expression of MMP-13 via the αvβ3 integrin, FAK, ERK, and NF-κB signal pathways (144). In summary, CTGF causes synovial fibrosis by activating fibroblast and their transition into myofibroblast, stimulates the production of pro-inflammatory cytokines and thus promotes synovitis and catabolic, destructive processes in the articular cartilage of OA patients.

### 5.3 Mechanical stress-mediated fibroblast activation

**Figure 7:**
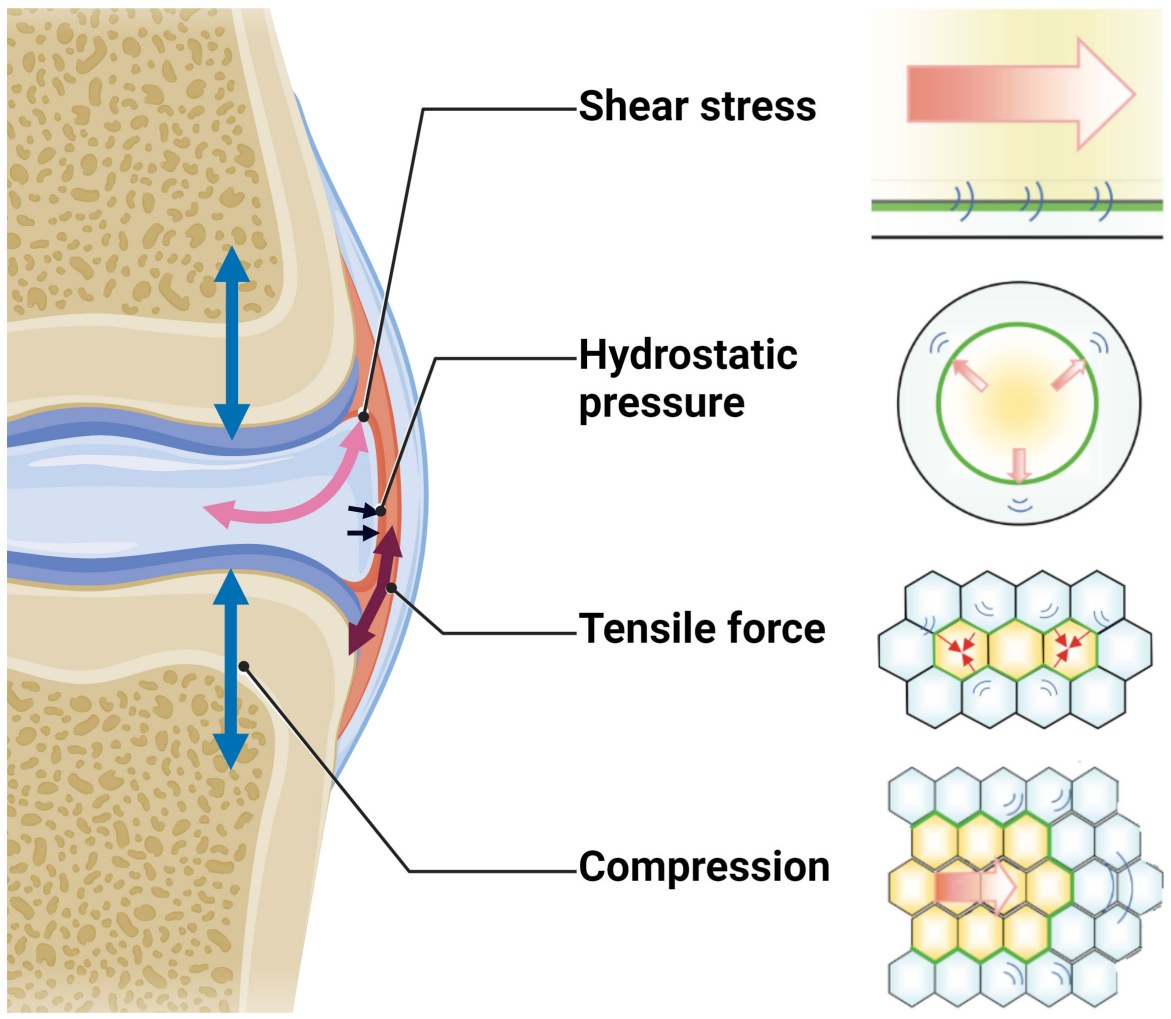
Schematic overview of the different mechanical forces that physiologically act on the joint during locomotion. Figure was created with BioRender.com with elements from (148).

The binding of cytokines and growth factors such as TGF-β or CTGF to their respective receptors is the predominant way of transmitting a signal and triggering signaling cascades in the responding cell, while electrical and mechanical stimuli also play an important role in signal transmission in a rather non-selective manner (145). In this way, mechanical forces are capable to regulate downstream signaling within the TGF-β pathway at multiple levels and influence both gene and protein expression (146). Additionally, myofibroblast contraction, tissue stiffening and aberrant tensile forces in the ECM activate latent TGF-β from ECM stores, thereby inducing e.g., Smad2/3-signaling and contributing to fibrosis (147). Beyond tensile forces, cells also respond to other forms of mechanical stimuli including compression, shear stress, and hydrostatic pressure (**Figure 7**) (148).

In joint tissues, shear and compression are the predominant mechanical forces. However, tensile forces notably influence the joint tissues as well. The sinister combination of high levels of pro-fibrotic factors such as TGF-β and CTGF in osteoarthritic joints with short- and long-term pathologically high mechanical forces facilitate the differentiation of FLS into myofibroblasts ─ a hallmark of OA (149–152).

While most research in OA in recent decades has focused on the role of cartilage and subchondral bone, synovial tissue and fibroblasts are gaining increasing attention. They are, in particular, recognized for their role in maintaining cartilage homeostasis and mitigating “wear and tear maintenance” (153). The gathered research demonstrates a nuanced correlation between mechanical stimuli and TGF-β signaling regulating various cellular pathways (**Table 2**). Upregulation of *COL1A1* and *FN1* genes in OA FLS and elevated levels of pro-inflammatory markers such as *PGE2*, *IL6*, and *IL8* in mechanically loaded samples demonstrate the interchangeable influences of mechanical forces and TGF-β signaling (154, 155). Cyclic strain on FLS elevates their *TIMP1* levels, while *MMP1* and *MMP13* appear to be diminished, suggesting regulation of ECM dynamics (157). *PRG4* expression, which plays a role in lubrication, is also induced and is further enhanced by TGF-β stimulation (159). Furthermore, mechanical stimulation of FLS increases the expression of *TGFB1*, with the resulting conditioned medium increasing pro-inflammatory markers in untreated FLS (161). Lysyl oxidases, crucial for collagen and elastin cross-linking, are generally increased after 6 h of physiological stretch (except LOXL-3) and decreased after pathological stretch (except LOXL-2). Here, a distinct temporal profile is evident, with a maximum of expression around 2-3 h and variations noted under differing stretching conditions (162). While some observations, such as IL-1α-mediated mechanosensitivity, are not analogous to TGF-β effects, they may contribute to the complexity of the fibroblast response to mechanical and biochemical stimuli (158). **Table 2** summarizes the mechanical stimuli applied to synovial fibroblasts, their intensity and mode of application, their effects, and any analogies to TGF-β signaling.

**Table 2:** Application of various mechanical stimuli on synovial fibroblasts, including types of forces, application methods, time intervals, and observed effects.

Mechanical stimulus	Force and application	Time interval	Cell type	Effects/TGF-β analogy (indicated by *)	Ref’s
Compression	2 g/cm^2^ with a glass disk	48 h	OA FLS	COL1A1 and FN1 upregulation in OA fibroblasts*, additionally increase of pro-inflammatory markers	(154)
Cyclic compression	40 kPa at 0.5 Hz with a cyclic load stimulator (CLS-5J-Z, Technoview, Japan)	1 h followed by 6 h incubation	FLS in a collagen matrix	PGE2, IL-6, and IL-8 upregulation in loaded samples, no differences in IL-1β and TNF-α expression*	(155)
Cyclic mechanical stretch	6% at 1 Hz in a FX-4000 Flexercell Tension Plus System (Flexcell Inc., USA)	2 h	RA and non-RA FLS	Physiological stretch decreases RA FLS proliferation (* context-dependent)	(156)
Cyclic strain	Peak-to-peak compressive replacement, equivalent to 2% axial strain	1 h at 6 rev./min	RA synovial cells	TIMP1 increase*, downregulation of MMP1 and MMP13 (*)?	(157)
Fluidic shear stress	0.5 dyn/cm^2^ in a parallel plate flow chamber	2 min	Bovine FLS on gelatine-coated glass slides	Mechanosensitivity mediated by IL-1α	(158)
Intermittent hydrostatic pressure	100 kPa in a custom pressurized stainless-steel chamber (Dentaurum Corporation, Germany)	4 h/day for 48 h	Murine non-OA FLS	Induction of PRG4 expression*, further increase with TGF-β stimulation	(159)
Static and dynamic stretching	16% (static), 10% (dynamic, short), 2% and 15% (dynamic mixed), and 15% (dynamic advanced); the static strain was applied through bioplex plates and a silicone stamp, dynamic strain in a custom-made cell stretcher	48 h, 4 h, 48 h, 48 h	Non-OA FLS	Decrease of COL1A2 expression in static stretching and no effects in short and mixed dynamic stretching with increase in advanced stretching*, increase of pro-inflammatory markers	(160)
Stretch	6%, 10% (with or without OA-conditioned media) in an Automated Cell Stretching System (STB-1400-10; Strexcell, USA)	24 h	OA FLS in collagen type I coated silicone chambers	Higher TGFB1 expression*, with conditioned media, higher pro-inflammatory markers	(161)
Stretch	6% (physiological) and 12% (injurious) in an equi-biaxial stretch chamber (Country Machines and Plastics, USA)	1 h, 2 h, 3 h, 6 h	Non-OA FLS	Upregulation of LOXs except LOXL3 after physiological stretch* further upregulation of LOXs between 1-3 h* and decrease after 6 h injurious stretch; decrease of MMPs at 6%, upregulation at 12%	(162)

Asterisks indicate analogies to TGF-β stimulation.

However, a critical limitation of the studies performed on synovial fibroblasts exposed to mechanical stimuli is the lack of studies on the key molecule α-SMA, which is encoded by the *ACTA2* gene and is a well-documented marker for myofibroblast differentiation to underpin the impact of mechanical strain on fibrosis.

### 5.4 Persistent inflammation and innate immunity drive fibroblast activation

Acute sterile inflammation is usually a transient and controlled response aimed at eliminating the cause of the injury and promoting tissue repair. However, if inflammation persists and becomes chronic, this well-controlled process can be pathologically disrupted (163). Inflammatory stimuli and innate immune responses play a central role in the development of OA, leading to chronic inflammation and tissue remodeling closely associated with the development of fibrosis. In OA, synovitis is a feature in the early stages and is characterized by the release of pro-inflammatory cytokines such as TNF-α, IL-1β, and IL-6 (164). Cytokines and other inflammatory mediators initiate cascades that contribute to the perpetuation of inflammation, synovial hyperplasia, hypoxia, subsequent synovial angiogenesis, progressive joint degradation, the activation of fibroblasts and their transition to myofibroblasts. Inflammatory cytokines contribute directly to the activation of synovial fibroblasts and promote the recruitment and activation of immune cells that further enhance inflammatory and fibrotic responses (165). Current studies do not provide evidence to conclude whether fibrosis can occur independently of synovitis, so it is not yet entirely clear which is the hen and which is the egg.

Usually, the early host defense of acute inflammation against invading pathogens implicates the recognition of conserved pathogen-associated molecular patterns (PAMPs) and damage-associated molecular patterns (DAMPs) (165, 166). The latter include calcium phosphate crystals, hydroxyapatite crystals, and uric acid found in the knee and hip joints of patients with OA. PAMPs and DAMPs are recognized by the pattern recognition receptors (PRRs) (167). and activate mechanisms of NLRP3 inflammasome activation leading to the production of inflammatory cytokines such as TNF-α and pro-IL-1β. Three prominent PRRs have been discovered, namely TLRs, nucleotide-binding oligomerization domain–like receptors (NLRs), and intracellular retinoic acid–inducible gene I receptors (RIG-I) (168, 169). They are present in various cell types, including immune cells and structural cells such as fibroblasts. Fibroblasts express a repertoire of PRRs, including TLRs, NLRs and IL-1R. For instance, DAMPS can activate mechanisms of NLRP3 inflammasome activation which trigger an innate immune response profile (167). This includes the production of numerous pro-inflammatory cytokines such as TNF-α and pro-IL-1β and chemokines which induce signaling cascades that promote the differentiation of FLS into myofibroblasts via auto- and paracrine signaling (169). Ospelt et al. compared FLS derived from trauma, RA, or OA patients, demonstrating constitutive gene expression for TLR1-6 but not for TLR7-10 (170). The *TLR3* and *TLR4* were the most abundant among TLR genes expressed in FLS, followed by members of the TLR2 subfamily. TLR2/4-signaling can be activated by hyaluronan fragments of distinct sizes which usually interact with CD44, a receptor for hyaluronic acid and other ligands, such as collagens, and MMPs (171, 172). As a result, a cellular pro-angiogenic and immunostimulatory response is initiated. Moreover, ED-A of FN, an endogenous TLR4 ligand, triggers the upregulation of MMP-9 supporting fibrosis (173, 174). Abdollahi-Roodsaz et al. showed that overexpression of IL-1β ─ a key cytokine in OA pathogenesis ─ triggers TLR4-mediated synovitis, whereas TLR2 plays a less significant role (175). Moreover, the severity of IL-1β-induced joint degradation in TLR4^-/-^ mice was reduced, with similar levels of inflammation, suggesting independent processes (175). Therefore, interfering with TLR signaling may be an approach to limit the activation of the FLS-mediated innate immune response, reducing inflammation, and limiting fibrosis.

Besides TLR-mediated FLS stimulation, FLS stimulation with pro-inflammatory cytokines such as TNF-α and IL-17A caused an increase in matrix mineralization, thereby inducing the expression of Wnt5a (176). Canonical Wnt/β-catenin signaling and inflammatory processes are strongly activated in OA synovium in mice and humans (177, 178). Using a PTOA mouse model, Knights and colleagues revealed that the Wnt/β-catenin signaling agonist *Rspo2* was strongly upregulated after injury and exclusively secreted by Prg4^+^ lining layer FLS (73). In rats with collagen-induced arthritis, activation of the Wnt/β-catenin signaling pathway was triggered by TNF-α, leading to a concomitant upregulation of α-SMA and cadherin-11 (CAD-11) expression in synovial fibroblasts (179). This subpopulation was found predominantly in the synovial lining layer in RA and was associated with high expression of PDPN (180). The strongest expression of PDPN^+^ FLS is also present in the lining layer in OA (181), suggesting a myofibroblast-like α-SMA^+^ ED-A-FN^+^ CAD-11^+^ PDPN^+^ phenotype. In addition, XAV-939, an inhibitor of the Wnt/β-catenin signaling, decreased the proliferation of human OA FLS and further inhibited the synthesis of collagen type I (178). This underscores the importance of the canonical Wnt/β-catenin signaling pathway in OA progression, which ultimately leads to synovial fibrosis in which the lining layer appears to be predominantly affected.

### 5.5 Cellular aging and its causes in osteoarthritis

The onset and progression of OA are closely linked to the cellular aging process in which cells accumulate in a state of senescence (182, 183). These senescent cells (SnCs) are characterized by permanent growth arrest, enhanced ROS synthesis, resistance to apoptosis, and a permanent low-level release of pro-inflammatory molecules. This phenotype is known as senescence-associated secretory phenotype (SASP). Removal of SnCs by administration of UBX0101(a compound inducing apoptosis in SnCs) in transgenic mice with anterior cruciate ligament transection (ACLT)-induced OA, inhibited cartilage erosion, reduced pain, and decreased inflammatory markers such as *mmp13* and *il1β*, and enhanced the number of ki-67-positive synovial fibroblasts (184).

While senescence’s impact on OA has been extensively studied in chondrocytes, its role in synovial fibroblasts and myofibroblasts still remains unclear (182, 185). In pulmonary fibrosis, the senescence of fibroblasts is evident (186), while senescence of other cell lineages such as macrophages, chondrocytes or cardiomyocytes have been related to various aging associated pathologies such as OA or cardiomyopathy (184, 187). Recently, Chen et al. demonstrated that FLS which are associated with impaired autophagy, show a SASP and accumulate in OA (188). These OA FLS express GATA binding protein 4 (*GATA4*), a regulator of cellular senescence, which is linked to increased expression of *p16INK4a*, *p21* and a SASP. Restoration of autophagy in OA FLS by rapamycin treatment was effective in reducing the expression of *GATA4* (188). Impaired autophagy (and mitophagy) leading to an accumulation of dysfunctional mitochondria and excessive ROS production (189). Chronic inflammatory diseases such as OA are characterized by elevated oxidative stress and ROS production (190, 191). Excessive ROS production is a major contributor to mitochondrial damage and dysfunction and to genomic damage as a cause for the onset of cellular senescence (189). In addition, mitochondrial dysfunction has been observed in the development of synovial fibrosis in OA (192). Mitochondrial ROS (mtROS) represent by-products of (patho)physiological processes with profound cellular consequences, ranging from modulation of intracellular signaling cascades to posttranslational modifications. The primary source of superoxide (O_2_^─^) in the cell, which is a product of NAD(P)H oxidase, arises from electron losses at complexes I and III in the electron transport chain (ETC). In various cell types, ROS formation is enhanced in response to TGF-β1 (193–195). These observations are of great importance for fibrogenesis, as fibroblasts are not only responsible for the generation of ROS, but ROS are also directly associated with the transformation of fibroblasts into α-SMA expressing myofibroblasts. A study conducted by Jain and colleagues demonstrated that the elevated mtROS generated at complex III are required for TGF-β-induced gene expression (193). In addition, the transcriptional activation of NADPH oxidase 4 (NOX4) by TGF-β requires the generation of mtROS. Notably, TGF-β-exposed fibroblasts derived from patients with pulmonary fibrosis exhibited higher mtROS production and increased pro-fibrotic gene expression compared to healthy subjects. Noteworthy reductions in TGF-β-induced pro-fibrotic gene expression and *NOX4* expression were observed upon the inhibition of mtROS production (193). These findings underscore the crucial role of complex III generated mtROS in TGF-β-mediated fibroblast to myofibroblast transition and suggest potential therapeutic avenues.

A study by Bondi and colleagues showed that rat kidney fibroblasts stimulated *in vitro* with TGF-β induced the expression of α-Sma, Fn, Nox2, and Nox4 and increased NAD(P)H oxidase activity, leading to mtROS production (195). The siRNA-mediated reduction of Nox4 significantly reduced the expression of α-Sma and Fn. Inhibition of TGF-β receptor 1 blocked Smad3 phosphorylation, decreased TGF-β-induced NADPH oxidase activity, and reduced the expression of Nox4, α-Sma, and Fn. Furthermore, TGF-β stimulated the phosphorylation of ERK1/2, which was inhibited by blocking TGF-β1 receptor 1 or Smad3. ERK1/2 activation also increased α-Sma and Fn (195). These results demonstrate that TGF-β-induced transition from fibroblasts to myofibroblasts involves canonical TGF-β/Smad2/3 signaling, non-canonical TGF-β/ERK1/2 signaling, and ROS production via NAD(P)H oxidase. Guido and colleagues generated fibroblasts overexpressing the mitochondrial fission factor (MFF). This factor regulates the mitochondrial network by continuous fission expanding the interconnected network of mitochondria when expressed at physiological levels. Overexpression of MFF caused mitochondrial dysfunction, mitophagy and autophagy, intracellular ATP depletion, increased ROS production, L-lactate secretion, and increased expression of α-SMA (196). Thus, MFF-induced mitochondrial dysfunction shifts cellular metabolism to a catabolic state in fibroblasts inducing α-SMA a key marker of myofibroblasts.

## 6 Metabolic pathways in fibroblasts and myofibroblasts

**Figure 8:**
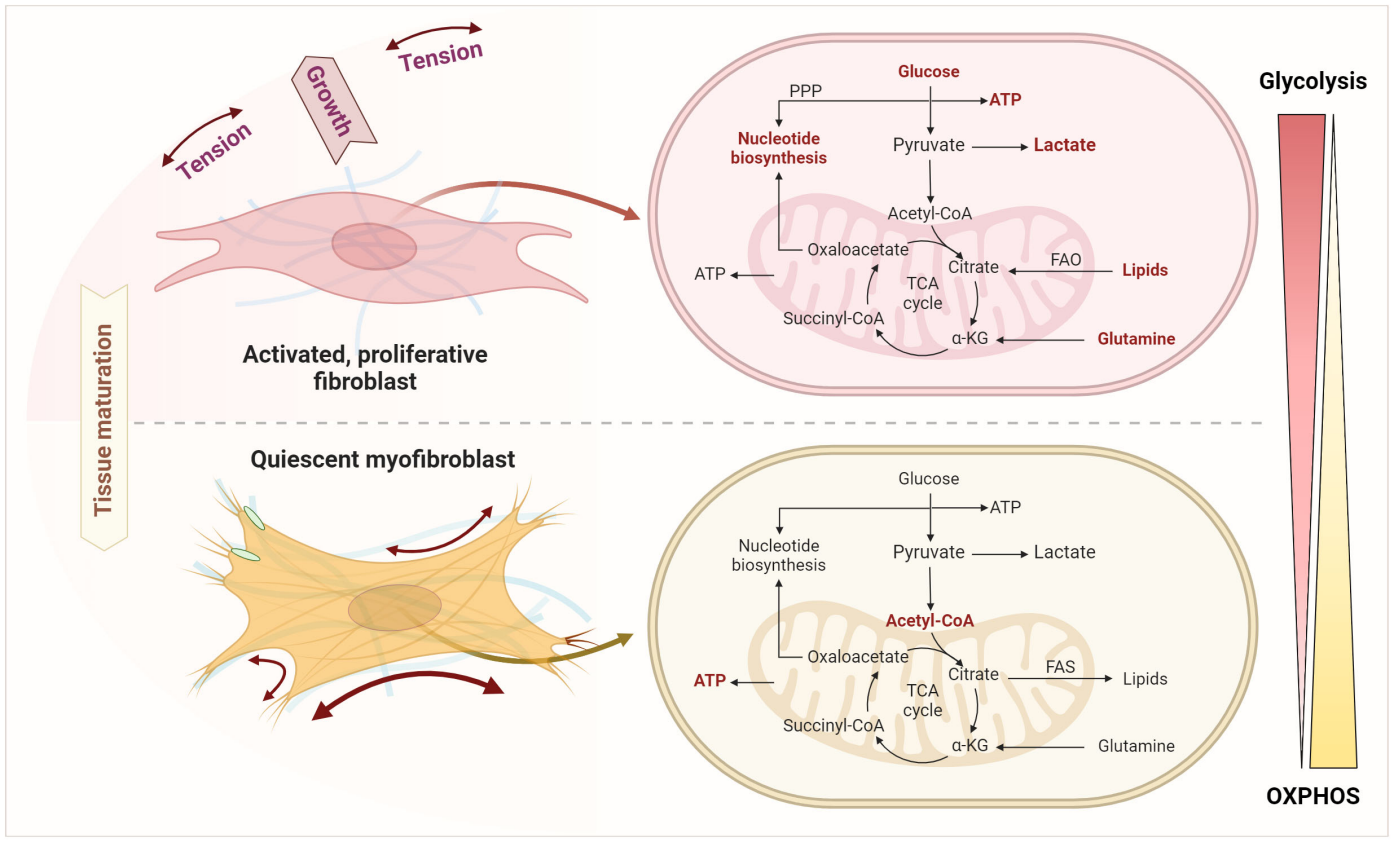
Metabolic shifts upon transition from fibroblast activation and expansion to senescent myofibroblasts. This transition involves an enhanced glycolysis, pentose phosphate pathway (PPP), fatty acid oxidation (FAO), lipolysis, and glutaminolysis as key processes in fibroblast activation and expansion. Quiescent myofibroblasts instead use glycolysis to generate acetyl-CoA fueling the TCA and mitochondrial oxidative phosphorylation to generate ATP and to feed lipid and fatty acid synthesis (FAS). Figure was created with BioRender.com.

In contrast to the extensively examined metabolic profile of chondrocytes in OA, little is known about the metabolic properties of FLS in OA (197). Previous studies on FLS metabolism primarily concentrated on FLS derived from RA patients. However, various stimuli, such as complement activation in the early stages of OA, pro-inflammatory cytokines, and in the later stages of OA a hypoxic environment, activate FLS. The transformation from fibroblasts to myofibroblasts is initiated by factors including cytokines, hormones, ligands (e.g., TGF-β, angiotensin II), ions (especially Ca^2+^), and mechanical forces (198, 199). The initial activation of fibroblasts leads to increased aerobic glycolysis, even in the presence of oxygen, leading to increased lactate production. This metabolic shift from oxidative phosphorylation (OXPHOS) in mitochondria, the primary ATP source, to aerobic glycolysis is known as the Warburg effect (**Figure 8**) (200). While most cells typically rely on mitochondrial OXPHOS for energy generation, glycolysis usually occurs in the absence of oxygen. The metabolic profile of initially activated fibroblasts differs from that of fully differentiated myofibroblasts. During fibroblast differentiation, a significant increase in ATP production is observed, as ATP is essential for the functionality of the contractile actin structure (198). Since fibroblast activation is accompanied by increased proliferation, synthesizing building blocks such as nucleotides and phospholipids is crucial. In contrast, myofibroblasts, representing fully differentiated senescent cells, are non-proliferative. Mitochondrial dysfunction, which leads to increased ROS formation and thus cell apoptosis and impaired energy metabolism ─ glucose, fatty acid, and glutamine metabolism ─ contribute to OA pathogenesis (201–203).

### 6.1 Glucose metabolism

Metabolic alterations in glycolysis play a central role regarding energy supply, biosynthesis, cell growth, and cell differentiation. Bardon, Ceder, and Kollberg were the first to demonstrate significantly increased activities of glycolytic enzymes such as hexokinase (HK), phosphofructokinase-1 (PFK1), pyruvate kinase (PKM) and lactate dehydrogenase (LDH) in activated fibroblasts from cystic fibrosis patients (204). The upregulation of numerous glycolytic genes that act as metabolic checkpoints has already been verified in various fibroblast populations. Aguilar and colleagues cultured rat cardiac fibroblasts in 5 mM glucose (normal) or 25 mM glucose (high) for 48 h (205). The results show that the O-GlcNAcylation of proteins and the protein content of TGF-β1 in cardiac fibroblasts increases with higher glucose concentrations, thereby activating pro-fibrotic signaling pathways and inducing an increase in collagen synthesis (205).

Assuming that extracellular glucose concentration alone is sufficient to induce fibroblast to myofibroblast transformation and thus fibrosis, Xie et al. inhibited glycolysis in lung fibroblasts by inhibiting 6-phosphofructo-2-kinase/fructose-2,6-biphosphatase 3 (PFKFB3) (206). In glycolysis, PFK1 is the first glycolysis-specific enzyme, a rate-limiting enzyme, and a key control point for regulating glycolytic flux. PFK1 is allosteric activated via the derivative fructose-2,6-bisphosphate, regulated by PFKFB3 (feedforward regulation). Therefore, inhibiting PFKFB3 prevented the differentiation of lung fibroblasts isolated from patients with IPF into myofibroblasts and attenuated the pro-fibrotic phenotype (206). Furthermore, upregulation of PFKFB3 was shown to be dependent on TGF-β1-activated Smad2/3, as both blocking the TGF-β1 receptor and silencing Smad2/3 abolished the induction of PFKFB3. Moreover, HIF-1α is downstream of increased glycolysis and represents a direct mechanism by which glycolysis is involved in pathologic myofibroblast differentiation (206). Activation of hepatic stellate cells (HSCs) is a key event during liver fibrosis. Since aerobic glycolysis is one of the metabolic hallmarks, Ban and colleagues investigated the effects of targeted inhibition on HSC activation (207). The glycolysis inhibitor 2-desoxy-D-glucose (2-DG) and costunolide showed similar effects in inhibiting the rate-limiting enzyme HK2, leading to decreased expression of HSC activation markers such as α-SMA and collagen I. This could be reversed by overexpression of HK2 induced by plasmid transfection, suggesting that inhibition of HK2 represents a new therapeutic option for (liver) fibrosis (207).

Another glycolytic enzyme that appears to be crucial for the formation of myofibroblasts is pyruvate kinase M2 (PKM2). PKM2 is an important factor in the Warburg effect of cancer cells (208) and can occur in two forms in proliferating cells such as fibroblasts: a catalytically highly active tetrameric and a less active dimeric form. While the tetrameric form is routing glucose metabolism to pyruvate and into the tricarboxylic acid (TCA) cycle for energy metabolism, the PKM2 dimer transfers the metabolic pathway to the pentose phosphate pathway (PPP), the uronic acid pathway (UAP), the polyol pathway (PYP), etc. for the material synthesis such as the synthesis of the subsequent five-carbon ribose and non-essential amino acids (209). Satyanarayana et al. analyzed the expression of PKM2 forms in cultured fibroblasts and myofibroblasts from tissues with fibrotic diseases (210). They showed that the less catalytically active PKM2 dimer is upregulated in myofibroblasts with a concomitant increasing NADPH production. This protects myofibroblasts from apoptosis, thereby feeding the *de novo* glycine synthesis needed for myofibroblasts’ collagen production and deposition. Moreover, conversion of the dimeric form to the catalytically active, tetrameric PKM2 form inhibited fibrosis progression in mouse models of liver, lung, and pancreatic fibrosis (210).

Smith and Hewitson showed that renal fibroblasts stimulated with TGF-β1 downregulate acetyl-CoA biosynthesis by inhibition of the pyruvate dehydrogenase complex (211). The increase in PDK1 in response to TGF-β1 stimulation corresponds to the increase in inactive PDH-E1α (211). Although lactate has traditionally been considered a by-product of the glycolytic pathway, recent studies suggest a possible direct pathophysiological role of lactate in the development of fibrosis. The study by Nho et al. showed increased lactate formation in fibrotic lung fibroblasts due to increased LDHA and a concomitant decrease in LDHB (212). LDHA converts pyruvate into lactate and NAD^+^, while LDHB is responsible for the conversion of lactate into pyruvate (213). High lactate concentrations lead to the conversion of lactate into pyruvate and the production of NADH, which results in an inhibitory feedback on glycolysis (213). In addition, lactate itself was shown to promote the differentiation of myofibroblasts into normal lung fibroblasts (from deceased individuals with no evidence of lung disease) via the lactate receptor GPR-81 (212). LDH5, one of the five isoenzymes of LDH, is most effective in catalyzing the conversion of pyruvate to lactate. In cancer cells, overexpression of LDH5 results in an increased glycolytic metabolism and a decreased need on oxygen. Consistently, Schruf and colleagues demonstrated that inhibition of LDH5 in primary lung fibroblasts attenuated TGF-β1-mediated metabolic switch to aerobic glycolysis (214). Nevertheless, LDH5 inhibition had no significant effect on TGF-β1-mediated transformation of fibroblasts to myofibroblasts in primary human lung fibroblasts, suggesting that the LDH5-dependent metabolic shift to aerobic glycolysis alone is not the decisive factor (214).

### 6.2 Fatty acid metabolism

The fatty acid metabolism, encompassing *de novo* synthesis, uptake, oxidation, and disposal of fatty acids, serves crucial roles at the cellular and organ levels. Fatty acid oxidation (FAO) is the preferred energy source for cells with a high metabolism. FAO generates more ATP than the oxidation of glucose, which is why it is necessary for the activation and proliferation of fibroblasts. In FAO, long-chain fatty acids are more easily absorbed via CD36. Medium-chain and short-chain fatty acids enter the cell without the need for specific transporters. Here, the FAO pathway is promoted by the upregulation of carnitine O-palmitoyltransferase 1 (CPT1) and peroxisome proliferator-activated receptor (PPAR) signaling. Nan and colleagues demonstrated that treatment with rosiglitazone, a ligand activating PPARγ, prevents the development of fibrosing steatohepatitis induced by a methionine-choline deficiency (MCD) diet in C57BL6/J mice (215). Furthermore, targeted overexpression of PPARγ by an adenovirus reduced the extent of liver fibrosis in male C57BL6 mice on MCD diet (216).

Basal autophagy is ubiquitously present in all cell types. It is rapidly upregulated as an adaptive response under cellular stress conditions to obtain intracellular nutrients and energy. In liver injury, it has been shown that autophagy is primarily upregulated in activated stellate cells while inducing fibrogenic markers. In this context, autophagy contributes to the intracellular degradation of lipids and correlates with increased fatty acid β-oxidation and mitochondrial OXPHOS (217). Hernández-Gea et al. demonstrated that HSCs from autophagy-deficient C57BL/6 mice were unable to process cytoplasmic lipid droplets, reducing the availability of free fatty acids (218). As a result, mitochondrial β-oxidation and ATP production were reduced, which attenuated fibrogenesis. Targeted inhibition of β-oxidation with etomoxir, simulating the effect of autophagy blockade, resulted in reduced expression of the fibrogenic genes *α-Sma, collagen 1α1, collagen 1α2, Pdgfr-β*, and *Mmp-2* (218). Obviously, blockade of autophagy is different to impaired autophagy and mitochondrial dysfunction since the later induces mtROS and cellular senescence also capable of inducing α-SMA expression supporting fibrosis (188, 192, 196). Conversely, glycolysis upregulation and FAO downregulation associated with higher lipid accumulation and accompanied by succinate accumulation has been demonstrated in fibroblasts and myofibroblasts from idiopathic pulmonary fibrosis (219). Furthermore, TGF-β1 induced succinate dehydrogenase (SDH) and succinate elevation. Elevated levels of succinate contribute to enhanced glycolysis and reduced FAO by stabilizing HIF-1α. Inhibition of SDH subunit A in fibroblasts prevents fibrosis formation and respiratory dysfunction deeming SDH as a new therapeutic target in fibrosis (219).

Moreover, acetyl-CoA carboxylase (ACC) catalyzes the rate-limiting step of *de novo* lipogenesis and regulates fatty acid β-oxidation. Using HSCs, Bates and colleagues investigated the role of ACC in liver fibrosis (220). Based on α-SMA expression and collagen production, they showed that inhibition of ACC reduces the activation of TGF-β-stimulated HSCs and thus reduces fibrosis. Inhibition of *de novo* lipogenesis also blocks glycolysis and induces FAO, demonstrating that *de novo* lipogenesis is required for fibroblast activation (220). It is known that the fibroproliferative effects of TGF-β are dependent on metabolic adaptation to maintain pathological growth. Herein, fatty acid synthase (FASN) is an essential anabolic enzyme in TGF-β-mediated fibrosis. Jung et al. showed that TGF-β increases FASN expression, which is mediated via the mammalian target of rapamycin complex 1 (mTORC1) (221). FASN expression correlated with the extent of pulmonary fibrosis in bleomycin-treated mice. Inhibition of FASN reduced the expression of pro-fibrotic targets and stabilized lung function, as analyzed by peripheral blood oxygenation (221). Metabolic comparison of synovial fibroblasts obtained from patients with RA, patients with OA, and seronegative controls showed that basal and maximal mitochondrial respiration was significantly lower in RA FLS compared to control FLS. In all donors, basal respiration was largely dependent on FAO, whereas glycolysis was highly utilized in FLS from RA patients (222). However, the evidence for the significance of FAO and FAS in OA and synovial fibrosis remains insufficiently understood and needs further research.

### 6.3 Glutamine metabolism

Glutamine is the most abundant amino acid in the human body. It plays an anaplerotic role replenishing the TCA cycle intermediates α-ketoglutarate (α-KG) during increased aerobic glycolysis and reduced OXPHOS (223). Fibroblasts stimulated with TGF-β show increased glutaminolysis, which contributes to increased α-KG content (224). The increased glutaminolysis can be achieved in fibroblasts by upregulation of the rate-limiting enzyme glutaminase 1 (GLS1) (224) and TGF-β (223). The study by Ge et al. found that increased glutaminolysis in myofibroblasts is required for collagen translation and stability (224). The amino acid composition of collagen is unique due to its high glycine content. Targeted inhibition of glutaminolysis with the Gls1 inhibitor CB-839 and BPTES, and genetic silencing by Gls1 short interfering (si)RNA, only reduced the expression of collagens, but not that of FN or α-SMA. This is consistent with the increased α-KG content in myofibroblasts, as α-KG activates mTORC1, which promotes the expression of collagen (224). Importantly, Bernard’s data from 2018 show that suppression of glutaminolysis after myofibroblast differentiation reverses TGF-β induced metabolic reprogramming (223).

Myofibroblasts are synthetic and contractile cells, and these functions are dependent on OXPHOS and ATP production. Glutaminolysis stimulated by TGF-β may therefore support myofibroblast functions by meeting bioenergetic needs by increasing OXPHOS and biosynthetic needs by providing anabolic carbons for e.g., FAS when succinate levels are high (219). Depletion of extracellular glutamine as well as silencing of GLS1 expression in the presence of glutamine prevented TGF-β induced myofibroblast differentiation (223). Studies showed that TGF-β induces the expression of the *de novo* serine [phosphoglycerate dehydrogenase (PHGDH), phosphoserine aminotransferase 1 (PSAT1) and phosphoserine phosphatase (PSPH)] and glycine [serine hydroxymethyltransferase 2 (SHMT2)] synthesis pathways in human fibroblasts (225). Genetic attenuation of PHGDH or SHMT2 and pharmacologic inhibition of PHGDH in human lung fibroblasts have shown that these enzymes are required for collagen synthesis downstream of TGF-β. Thereby, PHGDH is the first and rate-limiting enzyme in this pathway (225). Hamanaka and colleagues investigated whether inhibiting *de novo* serine and glycine synthesis mitigates lung fibrosis *in vivo* (226). TGF-β stimulation induces Phgdh expression at both mRNA and protein levels in mouse fibroblasts, and this effect is reflected by an increase in Phgdh expression in mouse lungs after intratracheal administration of bleomycin. However, treatment of murine and human lung fibroblasts with a small molecule Phgdh inhibitor (NCT-503) reduced TGF-β induced collagen protein synthesis. Moreover, mice treated with the Phgdh inhibitor seven days after intratracheal instillation of bleomycin exhibited attenuation of lung fibrosis (226). In summary, these results demonstrate that glucose-derived serine and glycine metabolism plays a pivotal role in the fibrotic response both *in vitro* and *in vivo*.

The transition from functional fibroblasts to fibroblast activation and expansion to senescent myofibroblasts involves several steps of metabolic alterations, highlighting glycolysis, FAO and glutaminolysis as key processes. This transition is critical in the development and progression of fibrotic diseases. Interventions targeting glycolysis, such as inhibition of PFKFB3, a key regulator of glycolytic flux, demonstrate potential in attenuating myofibroblast differentiation. Furthermore, Alterations in enzymes such as PKM2 and shifts in metabolic pathways (e.g., from TCA cycle to PPP) play a critical role in myofibroblast functionality and survival, thus influencing fibrosis progression. Similarly, FAO is essential for fibroblast activation. Targeting key metabolic enzymes in FAO such as ACC and FASN could provide promising anti-fibrotic effects. Moreover, TGF-β-induced metabolic reprogramming leads to increased glutaminolysis and serine/glycine synthesis, essential for collagen production and myofibroblast differentiation. Thus, targeting key enzymes such as GLS1 and PHGDH offers promising avenues for therapeutic intervention in fibrotic diseases. These findings emphasize the importance of metabolic pathways in fibrosis and the potential of metabolic interventions as therapeutic strategies.

## 7 Therapeutic strategies

Although we have gained more and more knowledge about the mechanisms of OA pathogenesis, there is still no effective cure. The treatment approaches for OA are different and depend on the joints affected (50). To date, they comprise physical, pharmacological, and surgical treatments (227). Physical treatment aims to reduce body weight, increase muscle strength, and reduce pain, e.g., through diet, exercises, physiotherapy or acupuncture (228–233). Standard in pharmacological treatment approaches do not yet prevent disability but aim to reduce symptomatic burden and pain of the disease and slow down tissue damage. For example, non-steroidal anti-inflammatory drugs (NSAIDs) including ibuprofen and acetaminophen, are effective as anti-inflammatory and analgesic drug. Glucocorticoids such as prednisolone are highly effective anti-inflammatory and immunosuppressive agents that primarily relieve synovitis in OA (234, 235). However, due to their pleiotropic effect, they also cause adverse clinical effects such as osteoporosis if not used carefully and responsibly (236). More specifically acting anti-cytokine drugs such as recombinant human IL-1ra counteract the pro-inflammatory, matrix-destroying effects of cytokines such as mediated by IL-1β (237). Chevalier et al. examined the safety of intraarticular injections of recombinant human IL-1Ra in patients with knee OA, which was well tolerated and did not induce any acute inflammatory reaction in OA patients (238). However, IL-1Ra treatment was not associated with improvements in knee pain, function, stiffness, or cartilage turnover (239). The authors argued that the lack of therapeutic efficacy could be due to systemic distribution or distribution outside the joint after intra-articular injection. Further studies with longer-acting and stronger IL-1 antagonists could lead to an improved clinical benefit.

In contrast, nerve growth factor (NGF) antibodies and opioids act on the reduction of pain (240). Anti-NGF antibody injections showed great effectiveness in reducing pain but also accelerated the development of osteoarthritis, if recipients were already taking NSAIDs (241–243). Chondroprotective agents, such as glucosamine, chondroitin sulfate and hyaluronic acid support lubrication and the viscoelastic properties of cartilage (244, 245). In addition, regenerative therapies with intra-articular injections of either platelet-rich plasma, MSCs, or Tissue Gene-C (TG-C) have reported positive results (246–248). While MSCs can self-renew, have immunomodulatory properties and a potential ability to differentiate into different cell lineages, TG-C is a cell-based gene therapy based on chondrocytes that are retrovirally transduced to overexpress TGF-β1. When conservative measures and medications are ineffective to alleviate the worsening of osteoarthritis, surgery arthroscopy to detect injuries of joints, repair injured soft tissues, such as ligaments and tendons, and bones, and remove inflamed and damaged tissue or artificial joint replacement is required.

Focusing on synovial fibrosis, we assume the transition from quiescent tissue resident FLS to activated FLS and finally myofibroblasts as a potentially initiating or contributing factor in OA pathogenesis. Based on the data reviewed, we hypothesize that micro lesions induced in the synovial membrane by mechanical stress initiate a vicious circle of inflammation, angiogenesis, regeneration, and fibrosis. This contributes to stress on the nervous system (pain), cartilage erosion and subchondral bone defects and instability of the ligaments and tendons further perpetuating the mechanical stress induced fibrotic synovitis. Notably, synovial fibrosis in OA seems to occur primarily in the lining and secondarily in the sublining layer, suggesting a mechanical stress-induced fibrosis. Several mechanisms in the fibrotic transformation of synovial fibroblast into myofibroblasts have been identified including the TLR engagement, NLRP3 inflammasome activation, pro-inflammatory cytokines (e.g., TNF-α, IL-1β), growth factors (e.g., TGF-β, CTGF), metabolic reprogramming (characterized by succinate accumulation, enhanced glycolysis, and glutaminolysis), hypoxia-induced HIF activation, and mitochondrial dysfunction leading to ROS generation and the SASP.

Some of the above strategies for managing OA are also aimed at treating synovitis and synovial fibrosis by interfering with e.g. TLR engagement, NLRP3 inflammasome activation, pro-inflammatory cytokines (e.g., TNF-α, IL-1β). Adalimumab, known for its efficacy in blocking the action of TNF-α, slows the progression of e.g., RA. The study by Verbruggen and colleagues investigated its potential effects on erosive hand OA (40 mg adalimumab or placebo) (249). After oral treatment, the disease appeared to remain active, as 40.0% of patients in the placebo group and 26.7% in the adalimumab group developed at least one new erosive interphalangeal joint in 3.6% and 2.1% of their non-erosive interphalangeal joints, respectively, during the 12-month follow-up period, demonstrating that adalimumab has no therapeutic effect on degenerative OA of the hand. The authors argued that the study design was not able to show effects of TNF blockade in individual finger joints as it aimed to measure overall hand function (249). Instead, neutralizing TNF-α significantly slowed down the progression of structural damage in already inflamed interphalangeal joints. Further studies are needed to confirm these results. Based on experience in the treatment of other fibrotic diseases, there are a variety of alternative intervention strategies that target the mechanisms in the fibrotic transformation of fibroblast into myofibroblasts such as small molecules or compounds that are currently in clinical trials demonstrating good anti-fibrotic properties (250). These anti-fibrotic drugs include receptor tyrosine kinase inhibitors, anti-TGF-β antibodies, TGF-β binding traps, TGF-β activation, inhibitors TGF-β receptor kinases inhibitors, JAK inhibitors, an anti-CTGF antibody, small molecule drug targeting β-catenin, and a TLR4 antagonist. In addition, altered metabolic programs might offer additional possibilities for novel therapeutic options (251).

Consequently, targeting pivotal signaling pathways and key molecules significantly influencing synovial inflammation and fibrosis in early-stage OA could offer therapeutic advantages. By interrupting the vicious cycle of damage-inflammation-dysfunctional repair at its early stages, there is potential for mitigating the progressive nature of OA and improving patient outcomes.

## 8 Conclusion

Osteoarthritis represents most complex whole-joint disorder involving multiple components including articular cartilage, subchondral bone, synovial fluid, synovial membrane, ligaments, tendons, adipose tissue, meniscus, and neurological routes. Pathologic features include pain, immobility, and inflammation. Despite extensive research, the pathogenesis of OA from onset to late-stage progression remains incompletely understood, highlighting the complexity of the disease’s underlying processes. It is widely accepted that risk factors include excessive mechanical loading, obesity, joint malalignment, prior joint injury, inflammation, aging, and various metabolic and genetic factors. These factors ultimately contribute to degradation of articular cartilage, inflammation and fibrosis of the synovium, irritation of the nociceptors (pain), aberrant angiogenesis of the synovium, disruption of subchondral bone, and instability of ligaments and tendons. These changes can play a role in the development and progression of the disease, either jointly or individually. Although synovial inflammation and fibrosis play crucial roles in OA pathogenesis, the exact mechanisms remain incompletely understood. Further research is needed to elucidate the interplay between synovial tissue, cartilage degradation, and joint inflammation in OA progression.

Furthermore, there is an urgent need for reliable biomarkers and imaging techniques that can detect OA at an early stage so that interventions can be carried out before irreversible joint damage occurs. Currently, diagnosis often happens in later stages when symptoms are pronounced and irreversible damage has already taken place. Identifying and characterizing specific fibroblast subsets associated with OA pathology could provide insights into the role of fibroblasts and their pathways. Translating these results into clinically relevant biomarkers and therapeutic approaches still remains a significant challenge. Bridging the gap between basic research and clinical practice requires rigorous validation of fibroblast-related targets and interventions in well-designed clinical studies.

## Author contributions

AD: Conceptualization, Project administration, Funding acquisition, Visualization, Data curation, Writing – original draft, Writing – review & editing. ER: Writing – original draft, Writing – review & editing. DA: Writing – original draft, Writing – review & editing. FB: Writing – original draft, Writing – review & editing. TG: Conceptualization, Project administration, Funding acquisition, Writing – original draft, Writing – review & editing.
